# Developmental Language Disorder as a Multidimensional Neurodevelopmental Spectrum: Implications for Diagnosis

**DOI:** 10.1044/2025_JSLHR-25-00089

**Published:** 2025-11-14

**Authors:** Karla K. McGregor, Lisa Goffman, Elena Plante, Krystal Werfel

**Affiliations:** a Center for Childhood Deafness, Language and Learning, Boys Town National Research Hospital, Omaha, NE; b Department of Speech, Language, and Hearing Sciences, The University of Arizona, Tucson

## Abstract

**Purpose:**

We aimed to create a comprehensive understanding of the dilemmas involved in the diagnosis of developmental language disorder (DLD) and to highlight the potential of a multidimensional spectral account of DLD for addressing these dilemmas.

**Method:**

We conducted an integrative literature review.

**Conclusions:**

Considerable gains in understanding the genetics and neurobiology of DLD provide new explanations for long-observed phenomena such as heterogeneity of presentation, phenotypic overlap with other neurodevelopmental conditions, and the challenges of diagnosis. Although categorical nosologies benefit diagnostic decision making, facilitate communication, and align well with bureaucratic systems, they offer overly simple ways of defining DLD. The presentation of DLD is broad rather than narrow, spectral rather than categorical, and dynamic rather than static; therefore, DLD is better described as a multidimensional spectral system than as a categorical entity. It is not practical or even advisable to abandon the traditional categorical framing of DLD at this point. However, maintaining awareness of the tension between the categorical framing and the spectral nature of DLD may improve diagnosis, prognosis, and alignment with the construct of neurodiversity.

… The present studies did not find evidence for a … taxon; instead, language skills in these children at these ages distributed in a dimensional rather than categorical fashion. These findings are consistent with other evidence about children with [developmental language disorder], who are heterogeneous with respect to severity and type of language deficits and often appear to exhibit more general deficits in other developmental domains. (Dollaghan, 2004, p. 470)

Developmental language disorder (DLD) is a highly prevalent *neurodevelopmental* condition that limits language learning, comprehension, and expression (Bishop et al., 2016, 2017; Calder et al., 2022; Norbury et al., 2016; Tomblin et al., 1997). As the term *neuro–* implies, the condition is brain-based. The neural substrates are not fully understood, but there is converging evidence of altered structure and function in the planum temporale, inferior frontal gyrus, and caudate nuclei (Abbott & Love, 2023). As the term *developmental* suggests, the condition emerges in childhood; however, DLD persists in adulthood, posing potential life-long challenges to social function, academic and professional success, and well-being (Conti-Ramsden & Botting, 2008; Dubois et al., 2020).

## How DLD Is Diagnosed

To diagnose is to categorize (Sonuga-Barke, 1998). When speech-language clinicians diagnose DLD, they are essentially saying that the observed symptoms and their severity are better classified as DLD than some other category (e.g., typical language development, speech sound disorder, autism spectrum disorder [ASD]). The diagnosis of DLD is based on behavioral data. Specifically, clinicians measure and describe language and communicative behavior via norm-referenced tests, spoken or written language samples, and responses to dynamic assessments. They also determine the persistence and functional impacts of the problem via observations, self-reports, or reports from caregivers and teachers (Bishop et al., 2016, 2017). They interpret these data in light of age-based expectations and the needs of the child and family (Kuiack & Archibald, 2024).

## Most People Who Have DLD Are Not Diagnosed With DLD

Despite the broad, long-term impact of DLD, many individuals go *undiagnosed* (Grimm & Schulz, 2014; Norbury et al., 2016; Tomblin et al., 1997). Of 488 children identified with DLD by Norbury et al. (2016), only 42.5% had been diagnosed and were receiving services. It is possible that some children had been diagnosed but were functioning well and thus did not need services; however, this is unlikely given that less than 12% of this group met grade-level academic expectations. The disadvantages of going undiagnosed are many. A diagnosis enables not only treatment but also access to information; self-understanding; connection with others who share that diagnosis; and, in some countries, accommodations and protection from discrimination (L. J. Graham & Tancredi, 2019; Tighe & Namazi, 2022).

Children with DLD are also at risk of being *misdiagnosed*. Because of symptom overlap, a child with DLD and social anxiety, for example, might be diagnosed with ASD (Williams et al., 2008), whereas a child with DLD who has difficulty following directions might be diagnosed with attention-deficit/hyperactivity disorder (ADHD; L. J. Graham, 2008). A serious disadvantage of being misdiagnosed is an ill-fitting course of treatment, including but not limited to unnecessary medication.

Finally, children with DLD are at risk of being *underdiagnosed*. As we will explore below, DLD frequently co-occurs with other neurodevelopmental conditions such as dyslexia, developmental coordination disorder, or ADHD. However, in some cases, receipt of the first diagnosis may discourage consideration of other potential diagnosis; in other words, signs of DLD may be overshadowed. Diagnostic overshadowing is a situation wherein certain aspects of a problem are overlooked in favor of those collectively confirming a particular preexisting diagnosis (Baraskewich & McMorris, 2019). The primary disadvantage of diagnostic overshadowing is that the treatment plan will be incomplete; it will address only some of the child's difficulties and, therefore, will provide limited improvement. “The most common reason why a therapy program is not working is that an important comorbidity (i.e., co-occurrence) is not being addressed” (Accardo & Shapiro, 2005, p. 248).

The goal of this review article is to advance understanding of the diagnostic dilemmas surrounding DLD. Without a doubt, part of the problem reflects low awareness of DLD (McGregor, 2020); however, we focus here on the fact that diagnosing DLD can be “fiendishly difficult” (Bishop, 2003, p. 309). We address this difficulty by (a) describing the nature of DLD at the behavioral, neural, and genetic levels; (b) summarizing the current diagnostic categorization of DLD; and (c) examining the tension between the nature of DLD and this categorization. In response to that tension, we will propose a multidimensional spectral account of DLD. In this account, a particular (and as yet not fully determined) system of dimensions defines DLD as a distinct diagnostic entity, while one or more, but not all, of these dimensions also comprise the defining system of other neurodevelopmental conditions. We will propose potential dimensions and suggest ways that a multidimensional spectral view of DLD informs best diagnostic practices. We will not argue for the abandonment of the traditional categorical framing, as it is not practical or even advisable to do so at this point in time. Rather, we aim to advance understanding of DLD and improve the services offered to individuals with DLD by reflecting on the limitations of this framing and the value of supplementing it with a spectral account.

## The Nature of DLD

### DLD Is a Neurodevelopmental Disorder

Neurodevelopmental disorders are a collection of conditions with onset in childhood that impede the development of one or more basic skills—talking, learning, interacting with others, thinking, or moving (Gillberg, 2010). The *Diagnostic and Statistical Manual of Mental Disorders, Fifth Edition–Text Revision* (*DSM-5-TR*; American Psychiatric Association, 2022), recognizes six broad types of neurodevelopmental conditions (see Table 1) and further indicates that any neurodevelopmental conditions may include an *associated specifier* if its biomedical, genetic, or environmental etiology is known (see also Bishop et al., 2016). So, for example, there are cases of intellectual disability associated with fetal alcohol syndrome, motor disorder associated with cerebral palsy, and language disorder associated with Landau–Kleffner syndrome. In the case of DLD, the specific etiological factors are as yet unknown; thus, no specifier is used.

**Table 1.** The Diagnostic and Statistical Manual of Mental Disorders–Fifth Edition nosology of neurodevelopmental disorders.

Intellectual disabilities
Intellectual disability (intellectual developmental disorder)
Global developmental delay
Unspecified intellectual disability (intellectual developmental disorder)
Communication disorders
(Developmental) language disorder
Speech sound disorder
Childhood-onset fluency disorder (stuttering)
Social (pragmatic) communication disorder
Unspecified communication disorder
Autism spectrum disorder
Attention-deficit/hyperactivity disorder (ADHD)
ADHD
○ Combined presentation ○ Predominantly inattentive ○ Predominantly hyperactive/impulsive
Other specified ADHD
Unspecified ADHD
Specific learning disorder
With impairment in reading
With impairment in written expression
With impairment in mathematics
Motor disorders
Developmental coordination disorder
Stereotypic movement disorder
Tic disorders
Other neurodevelopmental disorders (specified/unspecified)

### At the Behavioral Level, DLD Has a Predictable yet Variable Presentation

By definition, individuals with DLD present with difficulties in learning, comprehending, and expressing language, whether spoken, written, or signed. Their language abilities will appear similar to that of a younger peer, although some language domains will likely be less mature than others (Leonard, 2017). Difficulties with the formal aspects of language (phonology, word forms, morpho-syntax, grammatical relationships) are ubiquitous, so much so that theories of DLD consistently seek to account for them (Goffman & Gerken, 2023; Leonard, 1989; Plante et al., 2002; Rice & Wexler, 1996; Ullman & Pierpont, 2005; van der Lely & Pinker, 2014). That said, some individuals also have difficulties in the semantic domain, including the development of rich semantic representations (McGregor et al., 2002), semantic fluency (Hall et al., 2017; Weckerly et al., 2001), semantic inferencing (McGregor et al., 2024), and comprehension of figurative language (Nippold, 1985; Norbury, 2004). Pragmatic skills, such as introducing and maintaining conversational topics or responding to the questions posed by conversational partners, may be weak (Bishop et al., 2000), although the weakness is sometimes attributed to difficulties with comprehension and formulation at the semantic or grammatical levels rather than to actual limitations in the pragmatic domain (Rice et al., 2005).

Problems associated with DLD extend beyond the linguistic domain per se. Nonverbal cognitive functions are typically lower than those of their peers without DLD (Gallinat & Spaulding, 2014; Plante, 1998). Difficulties with memory, especially phonological short-term memory (e.g., Archibald & Gathercole, 2007; Marton & Schwartz, 2003) and verbal working memory (e.g., E. Jackson et al., 2020; Larson & Ellis Weismer, 2022; Montgomery, 2003), are common, as are problems with attention (Ebert & Kohnert, 2011; Smolak et al., 2020; Spaulding et al., 2008) and executive function (e.g., Kapa et al., 2017; Marini et al., 2020). Other symptoms may include difficulties with rhythmic timing (Fiveash et al., 2021), speed of processing (Abbott et al., 2024; Miller et al., 2001; Windsor & Hwang, 1999; Zapparrata et al., 2023), auditory processing (Barman et al., 2022; Bishop et al., 1999; Kujala & Leminen, 2017; Simpson et al., 2022), statistical (implicit pattern) learning (e.g., Evans et al., 2009; Lammertink et al., 2017; Obeid et al., 2016; Plante et al., 2013, 2017), and motor function (e.g., Hill, 2001; Sack et al., 2022).

The behavioral profile, or phenotype, is dynamic rather than static. It changes as the affected individual grows and develops and is faced with new expectations. In milder cases, the individual might eventually appear to outgrow the condition, but that is not typically the case. It is unusual to find remission of DLD after age 4 years (Bornstein et al., 2014; McKean et al., 2017; Norbury et al., 2017; Rice & Hoffman, 2015; Tomblin et al., 2003). When placed under high task demands, traces of the problem are discernable. For example, English-speaking children with DLD have difficulty marking tense in their spoken sentences when they are 4 years old, but that difficulty resolves by around age 8 years (Rice, 2013). However, those errors reappear when older children with DLD are asked to write rather than speak sentences (Scott & Windsor, 2000), to read aloud (Werfel et al., 2017), or to make metalinguistic judgments about the accuracy of tense marking (Rice et al., 2009). Similarly, morphosyntactic errors are relatively rare in the spontaneous speech of French-speaking children with DLD (Thordardottir & Namazi, 2007) but common and diagnostically relevant in their elicited productions (Franck et al., 2004) even into adolescence (Courteau et al., 2023). Although the presentation of DLD varies with the typology of the language (Leonard, 2000), a developmentally appropriate challenge reveals the persistent nature of the problem. It is essential to keep this in mind so that older children and adults with potential DLD do not go undiagnosed.

It is critical to note that phenotypic variation is expected, even when age is held constant. No two individuals with DLD will present in exactly the same way. One is likely to have a broader or more severe set of challenges than the other. Because of the broad and varying ways that DLD presents across the population and because other neurodevelopmental conditions also impact language learning, there are often partial overlaps between the language phenotypes that characterize DLD and those that characterize other neurodevelopmental conditions including ASD (Félix et al., 2024), dyslexia (Adlof et al., 2021), ADHD (Parks et al., 2023), auditory processing disorder (de Wit et al., 2018), and developmental coordination disorder (Archibald & Alloway, 2008). These overlaps complicate differential diagnosis.

**Figure 1.**
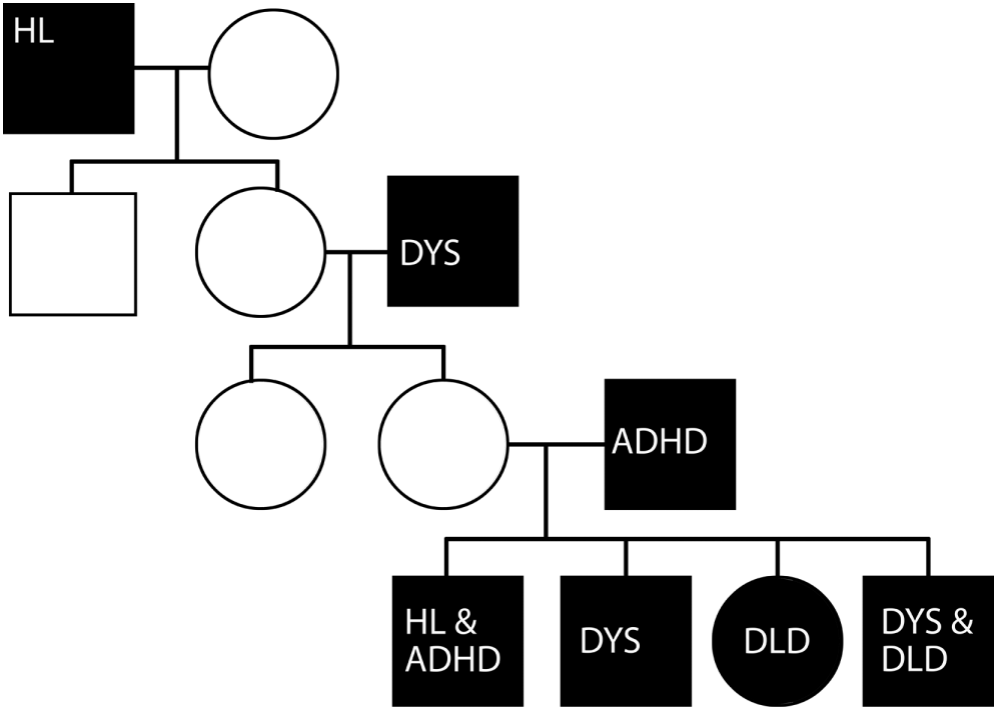
Neurodevelopmental disorders across multiple generations of a family, from oldest (top) to youngest (bottom). Squares indicate males; circles indicate females. Shaded shapes indicate affected individuals. Congenital hearing loss is not traditionally categorized as a neurodevelopmental disorder, and it is not known to co-occur with DLD at rates higher than would be expected in the general population. That said, difficulties parsing language problems associated with hearing loss from true cases of DLD may artificially limit such estimates. We include congenital hearing loss here because it reappears across generations, as does dyslexia and ADHD, in this particular family. In both cases, the congenital hearing loss was sensorineural. In the male from the youngest generation, it was unilateral. In the male from the oldest generation, it is not clear whether the congenital hearing loss was unilateral or bilateral. At the time of death, his hearing loss was bilateral. ADHD = attention-deficit/hyperactivity disorder; DLD = developmental language disorder; DYS = dyslexia; HL = congenital hearing loss.

In some cases, the breadth of presentation merits two or more diagnoses; in other words, DLD and some other neurodevelopmental condition(s) may affect the same individual. To understand why this happens, it may be useful to consider co-occurrences within the context of a family (see Figure 1). Neurodevelopmental disorders run in this family, but at the individual level, they manifest differently. Some individuals have one neurodevelopmental condition, some have another, and some have both. Within the youngest generation, one girl and one boy have DLD; the boy also has co-occurring dyslexia.

Within the individual, co-occurrences may be concurrent or successive (Dewey, 2018). As for the latter, consider that at 4 years old, a child might be diagnosed with both DLD and speech sound disorder (Deevy et al., 2010). At 6 years old, that same child may no longer show signs of a speech sound disorder but may be diagnosed with ADHD (Westerlund et al., 2002) and, at 10 years old, with a specific learning disability in writing (S. Graham et al., 2020), math, or reading (Young et al., 2002). This progression is logical, given the emergent nature of neurodevelopmental conditions and the need to diagnose them relative to age-based expectations. The possibility of successive co-occurrences holds implications for prognosis and continuity of care.

One should pay particular attention to signs of clinical conditions that co-occur with DLD at a prevalence that is higher than expected by chance. Estimates of the prevalence of any given co-occurrence will vary with (a) the diagnostic criteria used by the researchers, (b) the age of the population being sampled, and (c) the condition deemed the referent or “index.” To understand the importance of specifying the index group, consider that, among English-speaking 6-year-olds, the co-occurrence of DLD (defined by scores at the 10th percentile or lower on two or more language composite scores) among those with speech sound disorder (measured by the presence of age-inappropriate deletion, substitution, or distortion errors) is higher than the co-occurrence of speech sound disorders among those with DLD (Shriberg et al., 1999).

Some well-documented co-occurrences in the DLD population are listed in Table 2. These are likely to represent only a subset. Using an algorithmic phenotyping approach, Nitin et al. (2022) searched a large set of medical records, 3,631 for individuals with DLD and 18,155 for matched controls. They found 105 symptoms or conditions associated with DLD. In an independent sample of medical records, they replicated 17 of these. Some, like ADHD, motor coordination problems, and learning disorders, were no surprise given previous literature, but others, like dermatitis, conjunctivitis, and symptoms concerning nutritional and metabolic conditions, represent newer findings that require replication and explanation. The critical point here is that co-occurrences are frequent. We must take care to ensure that the diagnosis of a given neurodevelopmental condition does not overshadow, and therefore interfere with, the diagnosis of DLD.

**Table 2.** Neurodevelopmental conditions that often co-occur with developmental language disorder and their prevalence rate compared with the prevalence of the condition in the index group.

Co-occurring condition	Source	Age or grade	Rate of co-occurrence with DLD	Rate of condition in index group^a^
Speech sound disorder	Shriberg et al. (1999)	Age 6 years	For SLI: 5%–8%	3.8% (SLI + NLI + TLD)
			For NLI: 14%–18%	
Social–pragmatic communication disorder	Ellis Weismer et al. (2021)	Grade 8	30%^b^	11% (DLD + TLD)
				9% (TLD only)
Attention-deficit/hyperactivity disorder	Beitchman et al. (1989)	Age 5 years	34%–38%	12%
Dyslexia	Adlof et al. (2017)	7–9 years	54%	27% (DLD + TLD)
Reading comprehension deficits	Tomblin et al. (2000)	Grade 2	52%	10% (TLD only)
Spelling disability	Young et al. (2002)	Age 19 years	For SLI: 29.3%	7.1% (TLD only)
			For SLI & NLI: 40.3%	
Mathematics disability (dyscalculia)	Young et al. (2002)	Age 19 years	For SLI: 42.7%	11.5%–12.2% (TLD only)
			For SLI & NLI: 53.9%	
Developmental coordination disorder	Flapper and Schoemaker (2013)	5–8 years	32%	5%–6% (general population as summarized in the study of Flapper & Schoemaker, 2013)

*Note.* Here, we share only one relevant study per condition, selected in part because they estimated co-occurrence and index rates in the same way and from the same source to ensure like comparisons. The one exception is the estimate for developmental coordination disorder. DLD = developmental language disorder; SLI = specific language impairment; NLI = nonspecific language impairment; TLD = typical language development.

a Some investigators calculated the index rate based on the entire sample (those with DLD and TLD combined). Others reported the index rate based on the TLD sample only. Shriberg et al. (1999) and Young et al. (2002) separated the DLD group into those with average or above nonverbal IQ (SLI) and those with lower than average nonverbal IQ (NLI). Flapper and Schoemaker reported the index rate from the “general population.”

b Ellis Weismer et al. (2021) applied two definitions of SCD; this estimate is based on the “concomitant” definition which refers to “cases displaying significant pragmatic impairments with or without structural language disorder, using five scales from the Children's Communication Checklist–2nd Edition—four pragmatic scales plus the coherence scale” (p. 1240).

### Interim Summary

Thus far, we have demonstrated that the behavioral phenotype of DLD is variable both across developmental time and across individuals of the same age. The phenotype is also broad; it may include not only problems with grammar, phonology, and word forms but also semantics and, perhaps, pragmatics. It may include one or more problems in nonlinguistic domains as well. Given its breadth, the DLD phenotype often overlaps with the phenotypes of other neurodevelopmental conditions. In some cases, the phenotype is so broad that two or more diagnoses are necessary. Next, we turn to a summary of the neural and genetic bases of DLD where the source of the variation, breadth, and overlap will become immediately evident.

### DLD is Brain-Based

At the neural level, DLD is characterized by differences in the structure and function of the brain that emerge early in development. Unlike the focal damage to an otherwise healthy brain that often accounts for acquired language disorders, the neural differences associated with DLD are often bilateral and widespread, with the extent of spread varying from person to person.

**Figure 2.**
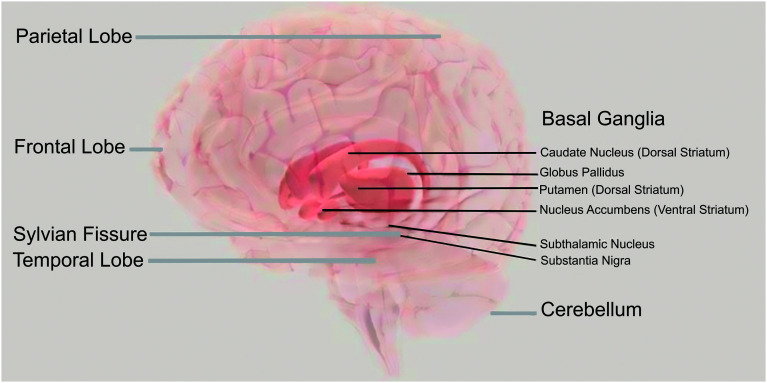
Neuroanatomical regions implicated in developmental language disorder: Cortical differences in the area around the Sylvian fissure radiating outward, subcortical differences in the basal ganglia, and differences in the connections between the two. The brain image is copyright of the Society for Neuroscience (BrainFacts.org, 2017).

*Cortical differences.* Long recognized as central to language functions in adults, it is not surprising that differences in the structure and function of the cortical regions surrounding the Sylvian fissure (also known as the lateral sulcus; see Figure 2) are consistently implicated in DLD (e.g., Badcock et al., 2012; Clark & Plante, 1998; Cohen et al., 1989; T. Jackson & Plante, 1996; Kurth et al., 2018; Plante et al., 1991, 2006, 2017). The Sylvian fissure divides the frontal and parietal lobes from the temporal lobe, and structural and functional differences also extend into these areas (Bahar et al., 2024; Krishnan et al., 2022; Pigdon et al., 2019; Ullman et al., 2024). Pigdon et al. (2019) also reported structural differences in the right cerebellum.

*Subcortical differences.* There is also increasing evidence for bilateral differences specific to the basal ganglia in individuals with DLD (Ullman et al., 2024). The basal ganglia are a collection of subcortical nuclei that connect directly and indirectly to numerous cortical regions. They are known to support the learning of rules, patterns, and habits, all of which are fundamental to language processing and production.

Krishnan et al. (2022) determined that children with DLD had reduced myelin but expected levels of iron in the caudate nuclei of the basal ganglia bilaterally. Conversely, Doucet et al. (2025) found that older children with DLD had higher iron content in the caudate (myelin was not considered), which was not the case in children with typical language development. Within their DLD group, higher brain iron in the caudate was associated with poorer narrative recall. The interpretation of this finding is complicated by an interaction with sex; girls with DLD showed a more substantial increase in iron with age than boys. Decisive conclusions about the precise differences in the structure and function of the caudate nuclei within the DLD population await future study.

*Combinations of cortical and subcortical differences.* We have separately summarized evidence of differences in the cortex and the basal ganglia among individuals with DLD. However, Krishnan et al. (2016) posit that the *combination* of developmental differences in the cortex and the striatal basal ganglia may account for DLD. Differences in one brain region alone may not be sufficient for DLD to present behaviorally. Consider, for example, that the siblings of children with DLD were found to have atypical reductions in the volume of the caudate nucleus of the striatum in one study (Badcock et al., 2012) and atypical perisylvian asymmetries in another (Plante, 1991), but these siblings did not present with DLD. Thus, a distributed pattern of brain differences may prove critical to the emergence of the condition.

An important caveat to the above summary is that the results refer to group-level differences; such differences do not characterize all individuals with DLD (Krishnan et al., 2022). Variation at the neural level may coincide with interindividual variation within the behavioral phenotype. Moreover, distributed patterns of brain differences characterize not only DLD but other neurodevelopmental conditions as well. In fact, some have questioned whether the goal of uncovering critical neural areas and networks involved in any specific neurodevelopmental disorder is feasible (Licari et al., 2019).

### Development Itself Shapes the Presentation of Neurodevelopmental Conditions

The brain is not highly specialized at birth but is shaped over time as the child experiences the world. In the neuroconstructivist approach, this notion is captured by the principle of context dependence. Context may be considered at multiple levels, two being the *cellular level*, where neural migration, neural differentiation, and neural survival are affected by cell-to-cell interactions in the brain, and the whole *brain level*, where neural connectivity emerges and is refined in a hierarchical progression (Sirois et al., 2008). Systems that support sensory motor processing develop first, and the systems that support language, cognition, and executive function are integrated later. The result is a highly interdependent system (Licari et al., 2019). Because of this interdependence, slight differences in neural development at one point can cascade to have larger effects over time (Karmiloff-Smith, 2006, 2009; Licari et al., 2019).

Neural development is further shaped by contexts at the *body level* where current capacities either enable or limit experiences and the *social level* where interactions with others promote adaptations and growth (Masten & Cicchetti, 2010; Sirois et al., 2008). To illustrate, consider that babies who are late to walk constrain their object word learning because they are limited in the ways that they can explore the physical context around them (Iverson, 2022; Karasik et al., 2014; Sanchez et al., 2018; Walle & Campos, 2014), and those who are late to talk change their social interaction and thus their opportunities for language learning (Hawa & Spanoudis, 2014; van Dijk et al., 2013). Thus, two different paths—being late to walk or being late to talk—could contribute to the emergence of language impairment. The variable phenotypic presentations of DLD as well as the overlaps between DLD and other phenotypes begin to make sense when one considers the idiosyncratic, dynamic, and cascading aspects of neural development.

### The Neural Differences Associated With DLD Result From Genes and the Contexts in Which They Are Expressed

Genomic variation is what makes each of us different from the other. Over one third of the genes in the human genome are active in the brain. The proteins produced by these genes contribute to the growth and development of brain structures, their interconnections, and functions (National Institute of Neurological Disorders and Stroke, 2024). Variations in (some of) these genes will contribute to neural differences that, in turn, contribute to the distribution of language abilities across human beings. Consider, for example, a meta-analysis conducted by Eising et al. (2022). From samples of saliva, buccal tissue, or blood, these investigators identified genetic variants associated with language performance (range of accuracy on language and literacy tasks). It turns out that these genetic variants help to determine the surface area of the superior temporal sulcus of the left hemisphere, suggesting a path from genes to brain to behavior.

Although some cases of DLD are attributed to variants in a single gene (e.g., *ATP2C2*, *CNTNAP2*, *NFXL1*), most cases of DLD are thought to be polygenic (Mountford et al., 2022). In these cases, a number of genetic variants, each of which may be relatively low risk, combine and interact to confer enough cumulative risk to result in DLD (Niemi et al., 2018). In a meta-analysis of the genetics of (developmental) language disorder, van Wijngaarden et al. (2024) reported a diverse group of 45 candidate genes. Within this set of genes, scientists may one day identify the complex combinations of genetic variations, gene-to-gene interactions, and gene–environment interactions that result in the neural signatures of DLD and, ultimately, its behavioral phenotype. Given the variability of the DLD phenotype, it could well be that there are multiple genetic paths to DLD.

Crucially, 22 of the 45 genes reported by van Wijngaarden et al. are also associated with other neurodevelopmental conditions, including intellectual disability, epilepsy, dyslexia, apraxia, and ASD. Thus, behavioral overlaps between DLD and these other conditions are not random; it reflects partially shared genetic bases (Kim & State, 2014). It seems that “specific mutations do not respect the boundaries of diagnostic categories” (Mitchell, 2012, p. 7).

### Interim Summary

We have made the case that DLD, like other neurodevelopmental conditions, does not make for a well-behaved category at the behavioral, neural, or genetic level. Different cases of DLD occupy different spaces within the category boundaries, and the boundaries themselves are fuzzy. Yet the clinician's job is to diagnose, and diagnosis involves categorization. The tension between the nature of DLD and the task of diagnosing it is obvious. We turn now to the frameworks that guide diagnostic decision making and the ways those frameworks help the clinician to address this diagnostic dilemma.

## DLD as a Diagnostic Category

### Current Nosologies

Clinicians in medical, allied health, and educational settings use decision rules to improve the validity and reliability of the identification of those who need services. These decision rules are sometimes formalized as diagnostic categories that reflect the medical tradition. Each category is assumed to capture a distinct type of impairment that is endogenous to the individual and common to all individuals who share the diagnosis (Sonuga-Barke, 1998). The *DSM-5* categorization of neurodevelopmental disorders presented in Table 1 is one such model. The *International Statistical Classification of Diseases and Related Health Problems–11th Edition* (*ICD-11*; World Health Organization, 2022) is another. However, these are broad nosologies that do not offer detailed decision rules for identifying DLD.

**Figure 3.**
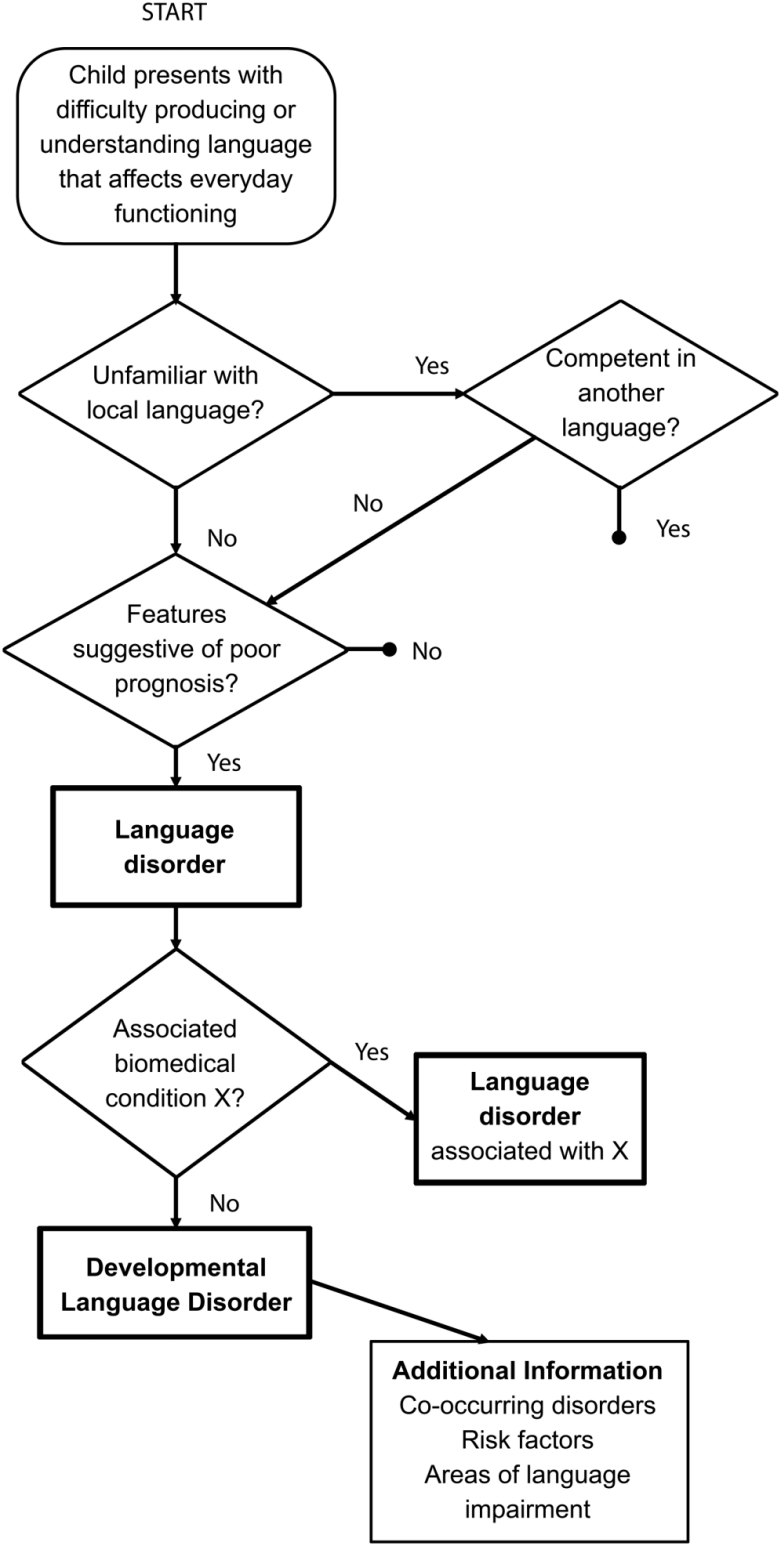
Proposed pathway to diagnosis of language disorder in Bishop et al. (2017). Original figure from Bishop et al. (2017).

A more specified diagnostic framework comes from the work of the CATALISE Consortium (Bishop et al., 2016, 2017). Their consensus recommendations, while compatible with the *Diagnostic and Statistical Manual of Mental Disorders, Fourth Edition*, go beyond it. Their overall guidelines are pictured as a flow chart in Figure 3. The first three levels of the flow chart are meant to “rule in” three defining characteristics of DLD: In a true case of DLD, the language problem is (a) severe enough to affect everyday (social, emotional, academic, or professional) function, (b) apparent in all languages being acquired, and (c) unlikely to resolve spontaneously. If these three characteristics are verified via interviews, observations, and behavioral testing including both static and dynamic assessments, then the individual has a language disorder.

The next level, common to the *DSM-5*, qualifies the condition by etiology. Specifically, if the individual also presents with a biomedical condition X (e.g., acquired conditions, neurodegenerative conditions, cerebral palsy, sensorineural hearing loss, ASD, intellectual disability) that likely accounts for the language problem, the diagnosis is not DLD but, rather, language disorder associated with X. These exclude DLD because they reflect complex impairments, including effects on language, and because some have neurological or genetic causes that do not apply to DLD. Whereas we endorse the CATALISE exclusionary criteria as a guide for current practice, we also note that there is no logical reason that a given individual could not have, for example, cerebral palsy and DLD. The one condition is not a vaccine against the other. Diagnostic nosologies are not static; they change as evidence emerges. As the field advances toward a more nuanced determination of what constitutes DLD, we may eventually be able to diagnose these co-occurrences.

There has been progress toward this end regarding deafness and DLD. Hawker et al. (2008) and Hardman et al. (2023) presented evidence of disproportionate language difficulty in some children learning spoken language via cochlear implants. They argue that these children have co-occurring deafness and DLD, and the additive effects of the two result in an unexpectedly high level of language impairment. Their hypothesis gains support in light of high rates of language disorder among the hearing siblings of cochlear implant users with severe unexplained language problems (Ramirez-Inscoe & Moore, 2011). In other words, language disorder may run in these families, affecting both the hearing and deaf siblings. The CATALISE Consensus group recognizes this possibility, pointing out that “some children have language abilities—in spoken and/ or signed language—that are well below those of their hearing-impaired peer group, and may be regarded as having a disproportionate language impairment that is not secondary to hearing loss” (Bishop et al., 2016, p. 16).

### How Shall We Account for Co-Occurrences in Diagnostic Nosologies?

Setting aside biomedical conditions that are likely to be causal, how shall we consider the high rate of co-occurring conditions associated with DLD? There are at least four possibilities. The estimated prevalence of any given co-occurrence may be inflated because co-occurrences are (a) artifacts of categorical distinctions that are too fine or (b) the result of imprecise diagnostic measures that fail to distinguish DLD fully from other conditions. On the other hand, the estimated prevalence of any given co-occurrence may accurately reflect the existence of (c) two or more separate categorical entities or (d) two or more spectral systems. We will use the co-occurrence of DLD and ADHD to illustrate each of these possibilities. As seen in Table 2, at age 5 years, the estimated rate of ADHD in the DLD population is about 3 times higher than in the general population (Beitchman et al., 1989).

The first possibility is that the high co-occurrence of DLD and ADHD is an artifact of categorical nosologies such as the *DSM-5* (First, 2005). These nosologies risk artificially defining boundaries between one condition and another that simply do not exist. In this view, many conditions we think of as co-occurrences could simply be a single condition with broad symptoms. Therefore, it is possible, for example, that ADHD is a condition that impairs both language and attention and differential diagnosis currently rests more on the relative salience of language versus attention rather than the presence or absence of either. To the extent that diagnoses drive treatment plans, one would want to be very conservative about broadening diagnostic boundaries.

A second possibility is that our current diagnostic measures are not precise enough to identify true distinctions between conditions. The work of Redmond et al. (2011) is notable here. They found that, among 7- to 8-year-olds, performance on a measure of narrative language ability did a good job of identifying those with DLD (sensitivity = .95) but a poor job at distinguishing it from ADHD (specificity = .65). In contrast, performance on a sentence recall task had both high sensitivity (.95) and specificity (.90). In other words, overlaps between these two groups seem greater when we evaluate them with narrative tasks (which tap a broad array of language abilities) than when we evaluate them with sentence repetition tasks (which rely more narrowly on memory and morphosyntax). To follow this line of thought through to its ultimate conclusion, if we could determine the right diagnostic measures, we might find that the co-occurrence between DLD and ADHD is no higher than expected given the rate in the general population.

A third possibility is the one represented in the CATALISE consensus statement (Bishop et al., 2016, 2017). That is to recognize that there are distinct disorders that do not explain DLD, but that tend to appear alongside it. These co-occurrences do not rule out the diagnosis of DLD (see final box of the flow chart in Figure 3). This recognition represents an advancement over earlier nosologies where the presence of ADHD, for example, would have prevented a DLD diagnosis (Stark & Tallal, 1981). Even when proposing their diagnostic nosology, Stark and Tallal (1981) noted that, in clinical practice, cases of “pure” language disorder were rare. The CATALISE recommendations do not remove the category boundaries between DLD and these other conditions, but they do acknowledge that they frequently co-occur.

The fourth possibility, and the one being developed here, is that category boundaries are, in fact, somewhat arbitrary but, at the same time, DLD presents as a separate diagnostic entity from other neurodevelopmental disorders. The lack of clear boundaries reflects partially but not fully shared underlying mechanisms (and hence partially but not fully shared phenotypes). The partial overlaps reflect interactions between language and other aspects of cognition (e.g., perception, memory, attention, action) in the emergence of DLD. Although some dimensions define both DLD and ADHD, the *system* of dimensions that define DLD is distinct from the *system* of dimensions that define ADHD. We shall return to this explanation of co-occurrences below when we discuss DLD as a multidimensional spectrum.

### How Shall We Account for Phenotypic Variability in Diagnostic Nosologies?

The heterogeneity of any given neurodevelopmental condition can complicate its diagnosis. A once-popular solution was to create subcategories to describe these variations. For example, in previous versions of the DSM, one could be diagnosed with classic autism, Asperger's, or pervasive developmental disorder not otherwise specified. In the *DSM-5-TR*, these subtypes are no longer included because, at the population level, there was no evidence for their validity (Frazier et al., 2012).

There is also a long history of efforts to define subtypes to account for heterogeneous presentations of DLD (Aram & Nation, 1975; Conti-Ramsden & Botting, 1999; Conti-Ramsden et al., 1997; Tomblin et al., 1997; van Daal et al., 2004; Van Weerdenburg et al., 2006; Wolfus et al., 1980). Despite these efforts, a subtype model of DLD has not held up over time. The proposed subtypes themselves are not particularly homogenous, leading Bishop to note that “systematic variation in the age and ability of children in different (DLD) subgroups suggest that these may correspond to variable manifestations of a core inherited language disorder, rather than distinct diagnostic entities” (Bishop, 1994, p. 1). Moreover, across studies, subtypes have not been reproducible, and many studies find no evidence for subtypes (Dollaghan, 2011; Lancaster & Camarata, 2019; Reilly et al., 2014).

### Interim Summary

To summarize, the diagnosis of DLD is guided by a categorical model in which conditions are construed as distinct. Such models assume that individuals who receive a given diagnosis have the same underlying impairment, even if it manifests somewhat differently from person to person. However, diagnostic nosologies are simplifications that do not necessarily reflect the true nature of the conditions categorized therein. They are only as accurate as the evidence base that informs their categorizations. As a result, nosologies that cover a broad range of diagnoses, as well as those that guide DLD diagnosis more specifically, change over time. We see signs that the condition we know as DLD sits uncomfortably within currently proposed category boundaries. In the next section, we propose an alternative framing of DLD, one that goes far toward accounting for co-occurrences and phenotypic variability.

## An Alternative: A Multidimensional Spectral Approach

The Research Domain Criteria (RDoC) project (Cuthbert, 2014, 2020) is a research framework that moves away from categorical models of mental disorders such as the *DSM-5* and the *ICD-11* and toward a multidimensional model of function that ranges from typical to impaired at the genetic, neural, and behavioral levels. The proposed dimensions (which are a work in progress) capture variations in traits across individuals and thus help to explain the heterogeneity we see within categorically defined conditions and the overlaps between those conditions. At the clinical level, the hope of the RDoC approach is that by identifying typical and impaired dimensions of function, rather than fitting profiles into categories, we will ultimately produce individually tailored treatments (Ibrahim & Sukhodolsky, 2021).

To apply a multidimensional framing to DLD, we begin with the foundational work of Tomblin and Zhang (2006). They examined the structure of the standardized language test outcomes of a large group of children followed and tested in Grades K, 2, 4, and 8 to determine the extent to which items purported to measure vocabulary versus sentence use and expressive versus receptive skills were measuring distinct constructs. They found no evidence that the development of expressive and receptive modalities was distinct at any grade. In other words, children's scores in one modality were strongly associated with scores in the other. They did find evidence that vocabulary and sentences were separate constructs, but only at Grade 8, suggesting that, initially, the lexicon and the grammar develop in tandem (see McGregor et al., 2005) and only gradually become distinct. Therefore, subtyping a child's DLD as “expressive” versus “receptive” or “grammatical” versus “lexical semantic” (during early development) is a practice that runs counter to current scientific evidence.

In a further exploration of the structure of DLD, Lancaster and Camarata (2019) tested three hypotheses: (a) Variations reflect membership in different subgroups (i.e., the traditional practice, not supported by Tomblin & Zhang, 2006), (b) variations are random individual differences (i.e., there are no discernable patterns), or (c) variations are spectral (i.e., predictable but not categorical). Using data from 505 kindergarteners with DLD in the Tomblin and Zhang sample, they applied clustering methods. Cluster analyses reveal whether there are any probabilistic patterns detected in a data set, not just the patterns that a researcher hypothesizes and, therefore, seeks to find. The results revealed nonrandom clustering, but the clusters overlapped and had little clinical validity. Instead, the patterns constituted substantial support for a spectral model of DLD. To date, the model itself is underspecified. Lancaster and Camarata (2019) point out the need to identify the dimensional traits that define the spectrum and, because they modeled data collected from standardized measures and in kindergarten only, determine whether and how those traits change over time. We turn there now.

### Toward Specification of a DLD Spectrum

**Figure 4.**
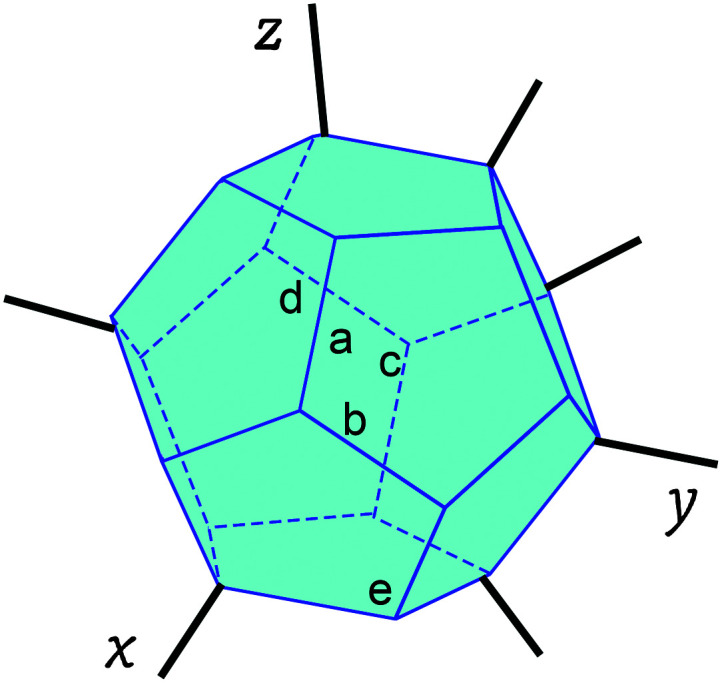
Developmental language disorder as a set of spectral traits (a, b, c, d, e) in a multidimensional space. Each ridge represents a trait that is characteristic of developmental language disorder (DLD). Each point of contact between ridges represents a potential interaction between traits. The multidimensional space that defines DLD will share one or more (but not all) ridges with other multidimensional spaces that define other neurodevelopmental conditions. Original figure from Mas et al. (2020).

Given the complexity of the DLD phenotype, a single spectrum represented by an invariant collection of language deficits that differ only in severity will not suffice. Instead, the spectral model of DLD is best represented as a multidimensional space (Astle & Fletcher-Watson, 2020), as depicted in Figure 4. Each ridge within that space is a potential defining trait that may or may not be affected in any given individual. Some but not all of these traits may also define other neurodevelopmental phenotypes.

Currently, there is no agreement on the precise defining traits of DLD. In Lancaster and Camarata (2019), the component traits comprised abilities measured by the subtests of standardized language and nonverbal cognitive assessments that were incorporated in the Tomblin and Zhang sample. These measured receptive and expressive vocabulary, receptive and expressive grammar, sentence imitation, articulation, narrative comprehension and recall, and visual design and pattern completion. Given the original intent of Tomblin and Zhang (2006), these subtests were chosen to capture a broad range of linguistic (and some nonlinguistic) abilities. For the secondary purpose of modeling the defining structure of DLD, they may be too broad. It is likely that children with DLD would show varying difficulties with all domains and that children with other language disorders would as well.

The extant literature suggests that one potential approach toward specification is defining traits derived from more precise measures. Accuracy of nonword repetition, sentence repetition, and judgments of tense and agreement markings have long been considered so sensitive to DLD as to constitute behavioral markers (Reilly et al., 2014; Rice & Wexler, 1996; Rujas et al., 2021). Moreover, these tasks sufficiently differentiate DLD from ADHD (Redmond et al., 2011), although the extent to which they differentiate DLD from other neurodevelopmental conditions is not as well established (Reilly et al., 2014). Another measure that shows promise is the number of words spoken per minute (Preza et al., 2025) or syllables spoken per second (Geng & Sheng, 2025). Both of these fluency measures are taken to reflect the effort involved in recalling or formulating spoken language. Each task, whether scored by accuracy or fluency, taps verbal short-term memory (especially nonword and sentence repetition) and grammatical knowledge (especially sentence repetition and tense and agreement judgments), suggesting that these are hallmark dimensions of DLD.

Another potential approach is to identify cross-domain behavioral co-occurrences and related neural functions that align with DLD. Ullman et al. (2020) hypothesized that domain-general deficits related to procedural learning underlie DLD. Goffman and Gerken (2023) present a more specific hypothesis in which aspects of the deficit are associated with sequential learning. These deficits are not random; instead, they reflect a higher-order cognitive variable associated with procedural or, more narrowly, with sequential learning. In this view, documented weaknesses, such as those observed in (a) verbal working memory, (b) processing speed, (c) language form (the learning and organization of relatively novel phonological and morphosyntactic elements), and (d) production of manual and musical sequences (Goffman et al., 2023; Kreidler et al., 2023), characterize DLD because they all derive from the processing of sequential dependencies. This theory-driven approach holds promise as it is more likely than language measures alone (or nonlinguistic measures that are not aligned with the weaknesses attested in DLD, such as aiming and catching [Sack et al., 2022]) to specify traits of DLD that do not fully overlap with those that characterize other NDDs. Notably, while there is a growing body of empirical support for relationships between nonlinguistic and linguistic features of DLD, to date, few clinical tools exist to assess procedural or sequential learning. As one example, implicit learning tasks, such as the serial reaction time task, are considered key measures of procedural learning but have not proven to result in reliable and replicable clinical measures (West et al., 2018).

Thus far, we have described three approaches for determining the dimensional traits that define DLD: test for a variety of language outcomes, use behavioral markers, and tap hypothesized domain-general underlying mechanisms. However, we have not addressed how these traits are to be weighted or how to consider their interactions. Working on problems of clinical psychology and psychiatry, Borsboom and Cramer (2013) and Borsboom (2017) view any given condition—let us call it neurodevelopmental condition A—as a system of traits. Critical to this view are the relationships between traits and the importance of those relationships in the emergence and maintenance of the condition. Specifically, any given trait may bear a direct or indirect causal relationship to another. These relationships comprise a system, and it is the system, rather than any individual trait, that differentiates neurodevelopmental condition A from neurodevelopmental condition B or C.

When applying this view to DLD, one might hypothesize, for example, that slow processing speed, weak attentional control, limited phonological memory, and poor sequential pattern learning establish and reinforce the condition that we recognize as DLD. The relationship between these traits at the system level distinguishes DLD from other conditions that might share one or more of the same traits, but not the entire system of traits. Heterogeneity within the DLD population could be accounted for in several ways in this approach. One possibility is that not everyone with DLD will have every trait, but each person will have key traits (or perhaps a critical number of traits) that merit the diagnosis. Another possibility is that all individuals with DLD share the entire system, but the relationship between any two traits within the system is stronger for one person with DLD than another. In either case, we can look to genetic, neural, and contextual factors as drivers of the variety of traits and their relationships.

An essential step forward is to determine how development changes the spectral traits and their interactions. Do the traits remain stable? How do we model developmental cascades? Do changing demands over developmental time readjust the weights within the system? It is not obvious how to embed the developmental timescale into the model (Borsboom, 2017). However, we can begin by working toward a more comprehensive description of the DLD phenotype in adolescents and adults. Knowing *what* to model is a prerequisite to knowing *how*.

We stand now on the precipice of a vast topic: the research agenda required for testing the validity of a multidimensional spectral view of DLD. Our aim here is to present and engage with some of the key issues that have both clinical and research relevance. However, for those readers who conduct behavioral research, we recommend the work of Astle and colleagues for a thoughtful overview of the changes needed in behavioral research. They lay out implications for the recruitment of participants, especially ways to ensure that phenotypic variability is not artificially reduced. They argue for the importance of transdiagnostic research studies and, consequently, the need to move from univariate to multivariate analytic approaches, which are better for addressing the complicated mixed samples involved in such research (Astle & Fletcher-Watson, 2020; Astle et al., 2022).

For those cognitive neuroscientists interested in DLD or other neurodevelopmental conditions, we direct you to Abbott and Love (2023) for their thoughts on how to move from exploratory to theory-driven neuroimaging studies. There are gains to be had from adding theory-motivated behavioral tasks to neuroimaging studies so that specific hypotheses can be tested. For example, if one proposes that phonological short-term memory problems are a defining dimension of DLD (as we do above), one would focus on networks that include the inferior frontal gyrus (implicated in phonological memory) and add behavioral tasks such as nonword repetition to determine whether any differences in the structure or function of the IFG correlate with performance. Moreover, in alignment with the argument made here, Abbott and Love encourage investigators to use narrow age ranges or to account for age in statistical models because of expected developmental changes. Comparing individuals of different ages likely contributes to the inconsistency that characterizes the literature on the neurology of DLD. Finally, they encourage the examination of distributed networks rather than isolated brain regions, and this too aligns well with notions of dimensionality and spectra.

### Advantages of the Multidimensional Spectrum Account

Although an account of DLD as a neurodevelopmental multidimensional spectrum is incomplete, the current conceptualization holds several immediate implications for changing clinical practice. But first, we will emphasize what does not change. The field is not ready to completely set aside the categorical model of DLD and perhaps never will be. The categorical model maps nicely onto nosologies that govern clinical practices such as billing (e.g., the *ICD-11* codes) and qualification for services (e.g., the disability categories in the Individuals with Disabilities Education Act, 2004). The inclusionary and exclusionary guidelines that follow from the categorical model improve the cross-clinician reliability of diagnosis. The standard nomenclature of the categorical model facilitates communication and understanding between all community members, from individuals with DLD and their families to the clinicians and educators who work with them to the researchers who study the condition (Bishop, 2003).

However, to optimize the diagnostic process, one must apply the categorical model while remaining fully aware of its assumptions and limitations (Sonuga-Barke, 1998). Pairing the categorical model with the neurodevelopmental multidimensional spectral model addresses some of these limitations. The spectral model may offer more nuanced diagnostic outcomes, a lower risk of diagnostic overshadowing, better prognostication, and better alignment with the construct of neurodiversity. We turn to these next.

*More nuanced diagnostic outcomes.* In categorical models, diagnoses are rigidly binary: One has DLD or does not. In contrast, a spectral approach guides the clinician to consider gradations of abilities and how subtle differences in abilities interact (Astle et al., 2022). Moreover, categorical models depend heavily on the identification of impairment, but those who hold to spectral models may be more motivated to identify dimensions of strength as well as dimensions of weakness.

*Lower risk of diagnostic overshadowing.* Categorical models applied to DLD have promoted—and to some extent continue to promote—a categorize-and-exclude approach: The child's language and communication problems must be DLD or something else. This faulty assumption may contribute to diagnostic overshadowing. Within a neurodevelopmental multidimensional framing of DLD, legitimate co-occurrences are less likely to be dismissed. Given partially overlapping genetic and neural causes, they may be expected. A clinician or a family member who does not understand this may seek a path of care that does not fully address the child's range of needs.

*Better prognostication.* Categorical models of DLD are not particularly useful for guiding discussions of prognosis, developmental change, and expected outcomes. In contrast, viewing DLD as a neurodevelopmental multidimensional spectrum allows meaningful consideration of these issues. The “developmental” nature of neurodevelopmental conditions should be emphasized in any discussion of the DLD diagnosis. Children with DLD will gain considerable skills as they grow up. At the same time, they may acquire additional diagnoses as developmental expectations change. This does not mean that the previous diagnosis was incorrect. Instead, with changing demands, symptoms of a co-occurring condition may emerge. Helping caregivers understand the high likelihood of improvement, the potential for presentations to change over developmental time, and the possibility that new diagnoses will apply in the future is crucial to their ability to understand and support their child, monitor for emerging challenges, and act accordingly.

*Better alignment with the construct of neurodiversity.* When counseling families and individuals who live with DLD, explaining DLD as a neurodevelopmental spectrum may be helpful, particularly to those who embrace notions of neurodiversity and neurodivergence. *Neurodivergent* refers to individuals who “ … process and engage in the world around them in ways that differ from the statistical or cultural expectation” (Sapiets, 2021). The first part of the term, *neuro*, emphasizes the role of brain development in these different ways of processing and engaging and thus readily applies to individuals with neurodevelopmental conditions, DLD included. The second part of the term, *divergent*, emphasizes the role of the external world (statistical and cultural expectations) in defining and identifying such differences and thus contrasts with the assumption that neurodevelopmental conditions are endogenous pathologies, an assumption that underpins categorical models from the medical tradition. Many of the characteristics of neurodevelopmental conditions, especially the role of context in their emergence and their dimensional nature, interindividual variation, and tendency to defy strict category boundaries, are highly compatible with notions of neurodiversity.

Diagnosing DLD does not, in our view, contradict the recognition that neurodivergent individuals, including those with DLD, “are tasked with navigating environments that are not optimized for their profile.” (Hobson et al., 2024, p. 3). In practice, integrating these ways of viewing DLD broadens treatment options. In some cases, the speech-language clinician might work to improve the person's ability; in others, to enable compensatory strategies; and in others still, to focus on improving the supports provided by the world around the person; in many cases, they will want to do all three.

## Summary and Conclusions

A full consideration of DLD as a neurodevelopmental spectrum has been a long time coming. Over 40 years ago, Stark and Tallal (1981) warned that the categorization of DLD as a condition specific to language was a forced fit. In the 10 years that followed, Aram et al. (1984), Bishop and Edmundson (1987), and Johnston and Smith (1989), among others, documented nonlinguistic deficits associated with DLD. Some 15 years later, Dollaghan (2004) warned of the harm inherent in structuring diagnostic practices around a forced and overly narrow categorization. Now, 20 years later still, we find ourselves listening to these founding mothers at a time when science has finally caught up with their observations. Considerable gains in understanding the genetics and neurobiology of brain-based disorders and associated behavioral features have prompted a rethinking of categorical diagnoses and provided alternative explanations for long-observed phenomena like heterogeneity of presentation, symptom overlap, and the challenge of differential diagnosis. These explanations motivate new diagnostic processes that establish strengths and weaknesses across a broad range of neurodevelopmental domains and enable the tailoring of interventions. Such processes require transdisciplinary training as well as health care and educational policy reforms to support and coordinate multiple care paths. For now, clinicians must balance the pragmatic benefits of categorical models with the knowledge that they are, at best, overly simple and, at worst, wrong. To paraphrase Graf (2020, p. 32), our goal must be to diagnose while recognizing the complexity and dynamics of the condition and the range of experiences belonging to each person with DLD.

## Data Availability Statement

Data sharing is not applicable to this article as no data sets were generated or analyzed during the current study.

## References

[bib1] Abbott, N., & Love, T. (2023). Bridging the divide: Brain and behavior in developmental language disorder. Brain Sciences, 13(11), Article 1606. 10.3390/brainsci13111606 PMC10670267 38002565

[bib2] Abbott, N., Nip, I., & Love, T. (2024). Rate of speech affects the comprehension of pronouns in children with developmental language disorder. Frontiers in Language Sciences, 3, Article 1394742. 10.3389/flang.2024.1394742 PMC11391914 39268499

[bib3] Accardo, J., & Shapiro, B. K. (2005). Neurodevelopmental disabilities: Beyond the diagnosis. Seminars in Pediatric Neurology, 12 (4), 242–249. 10.1016/j.spen.2005.12.006 16780295

[bib4] Adlof, S. M., Baron, L. S., Bell, B. A., & Scoggins, J. (2021). Spoken word learning in children with developmental language disorder or dyslexia. Journal of Speech, Language, and Hearing Research, 64(7), 2734–2749. 10.1044/2021_JSLHR-20-00217 PMC8632516 34185581

[bib5] Adlof, S. M., Scoggins, J., Brazendale, A., Babb, S., & Petscher, Y. (2017). Identifying children at risk for language impairment or dyslexia with group-administered measures. Journal of Speech, Language, and Hearing Research, 60(12), 3507–3522. https://pubs.asha.org/doi/abs/10.1044/2017_JSLHR-L-16-0473 10.1044/2017_JSLHR-L-16-0473 PMC5962925 29222567

[bib6] American Psychiatric Association. (2022). Diagnostic and statistical manual of mental disorders, Fifth Edition, Text Revision (DSM-5-TR®).

[bib7] Aram, D. M., Ekelman, B. L., & Nation, J. E. (1984). Preschoolers with language disorders: 10 years later. Journal of Speech and Hearing Research, 27(2), 232–244. 10.1044/jshr.2702.244 6738035

[bib8] Aram, D. M., & Nation, J. E. (1975). Patterns of language behavior in children with developmental language disorders. Journal of Speech and Hearing Research, 18(2), 229–241. 10.1044/jshr.1802.229

[bib9] Archibald, L. M., & Alloway, T. P. (2008). Comparing language profiles: Children with specific language impairment and developmental coordination disorder. International Journal of Language & Communication Disorders, 43(2), 165–180. 10.1080/13682820701422809 17852518

[bib10] Archibald, L. M., & Gathercole, S. E. (2007). Nonword repetition in specific language impairment: More than a phonological short-term memory deficit. Psychonomic Bulletin & Review, 14(5), 919–924. 10.3758/bf03194122 18087960

[bib11] Astle, D. E., & Fletcher-Watson, S. (2020). Beyond the core-deficit hypothesis in developmental disorders. Current Directions in Psychological Science, 29(5), 431–437. 10.1177/0963721420925518 33071483 PMC7539596

[bib12] Astle, D. E., Holmes, J., Kievit, R., & Gathercole, S. E. (2022). Annual research review: The transdiagnostic revolution in neurodevelopmental disorders. The Journal of Child Psychology and Psychiatry, 63(4), 397–417. 10.1111/jcpp.13481 34296774

[bib13] Badcock, N. A., Bishop, D. V., Hardiman, M. J., Barry, J. G., & Watkins, K. E. (2012). Co-localisation of abnormal brain structure and function in specific language impairment. Brain and Language, 120(3), 310–320. 10.1016/j.bandl.2011.10.006 22137677 PMC3315677

[bib14] Bahar, N., Cler, G. J., Krishnan, S., Asaridou, S. S., Smith, H. J., Willis, H. E., Healy, M. P., & Watkins, K. E. (2024). Differences in cortical surface area in developmental language disorder. Neurobiology of Language, 5(2), 288–314. 10.1162/nol_a_00127 38832358 PMC11093399

[bib15] Baraskewich, J., & McMorris, C. A. (2019). Internalizing mental health issues in individuals with neurodevelopmental disorders: Implications for practitioners. Current Developmental Disorders Reports, 6(1), 1–8. 10.1007/s40474-019-0154-9

[bib16] Barman, A., Prabhu, P., Mekhala, V. G., Vijayan, K., & Swapna, N. (2022). Auditory processing in children with specific language impairment: A FFR based study. Indian Journal of Otolaryngology Head and Neck Surgery, 74(Suppl. 1), 368–373. 10.1007/s12070-020-02127-x PMC9411455 36032839

[bib17] Beitchman, J. H., Hood, J., Rochon, J., & Peterson, M. (1989). Empirical classification of speech/language impairment in children II. Behavioral characteristics. Journal of the American Academy of Child & Adolescent Psychiatry, 28(1), 118–123. 10.1097/00004583-198901000-00022 2914824

[bib18] Bishop, D. V. (1994). Is specific language impairment a valid diagnostic category? Genetic and psycholinguistic evidence. Philosophical Transactions of the Royal Society of London. Series B: Biological Sciences, 346(1315), 105–111. 10.1098/rstb.1994.0134 7886145

[bib19] Bishop, D. V. (2003). Specific language impairment: Diagnostic dilemmas. In L. Verhoeven & H. van Balkom (Eds.), Classification of developmental language disorders (pp. 321–338). Psychology Press. 10.4324/9781410609021-21

[bib20] Bishop, D. V., Carlyon, R. P., Deeks, J. M., & Bishop, S. J. (1999). Auditory temporal processing impairment: Neither necessary nor sufficient for causing language impairment in children. Journal of Speech, Language, and Hearing Research, 42(6), 1295–1310. 10.1044/jslhr.4206.1295 10599613

[bib21] Bishop, D. V., Chan, J., Adams, C., Hartley, J., & Weir, F. (2000). Conversational responsiveness in specific language impairment: Evidence of disproportionate pragmatic difficulties in a subset of children. Development and Psychopathology, 12(2), 177–199. 10.1017/S0954579400002042 10847623

[bib22] Bishop, D. V., & Edmundson, A. (1987). Specific language impairment as a maturational lag: Evidence from longitudinal data on language and motor development. Developmental Medicine & Child Neurology, 29(4), 442–459. 10.1111/j.1469-8749.1987.tb02504.x 2445609

[bib23] Bishop, D. V., Snowling, M. J., Thompson, P. A., Greenhalgh, T., & CATALISE Consortium. (2016). CATALISE: A multinational and multidisciplinary Delphi consensus study. Identifying language impairments in children. PLOS ONE, 11(12), Article e0158753. 10.1371/journal.pone.0158753 PMC4938414 27392128

[bib24] Bishop, D. V., Snowling, M. J., Thompson, P. A., Greenhalgh, T., & CATALISE Consortium. (2017). Phase 2 of CATALISE: a multinational and multidisciplinary Delphi consensus study of problems with language development: Terminology. The Journal of Child Psychology and Psychiatry, 58(10), 1068–1080. 10.1111/jcpp.12721 28369935 PMC5638113

[bib25] Bornstein, M. H., Hahn, C. S., Putnick, D. L., & Suwalsky, J. T. (2014). Stability of core language skill from early childhood to adolescence: A latent variable approach. Child Development, 85(4), 1346–1356. 10.1111/cdev.12192 25165797 PMC4286341

[bib26] Borsboom, D. (2017). A network theory of mental disorders. World Psychiatry, 16(1), 5–13. 10.1002/wps.20375 28127906 PMC5269502

[bib27] Borsboom, D., & Cramer, A. O. (2013). Network analysis: An integrative approach to the structure of psychopathology. Annual Review of Clinical Psychology, 9(1), 91–121. 10.1146/annurev-clinpsy-050212-185608 23537483

[bib141] BrainFacts.org. (2017). 3D Brain. https://www.brainfacts.org/3D-Brain#intro=false&focus=Brain

[bib28] Calder, S. D., Brennan-Jones, C. G., Robinson, M., Whitehouse, A., & Hill, E. (2022). The prevalence of and potential risk factors for developmental language disorder at 10 years in the Raine study. Journal of Paediatrics and Child Health, 58(11), 2044–2050. 10.1111/jpc.16149 35922883 PMC9804624

[bib29] Clark, M. M., & Plante, E. (1998). Morphology of the inferior frontal gyrus in developmentally language-disordered adults. Brain and Language, 61(2), 288–303. 10.1006/brln.1997.1864 9468774

[bib30] Cohen, M., Campbell, R., & Yaghmai, F. (1989). Neuropathological abnormalities in developmental dysphasia. Annals of Neurology, 25(6), 567–570. 10.1002/ana.410250607 2472772

[bib31] Conti-Ramsden, G., & Botting, N. (1999). Classification of children with specific language impairment: Longitudinal considerations. Journal of Speech, Language, and Hearing Research, 42(5), 1195–1204. 10.1044/jslhr.4205.1195 10515515

[bib32] Conti-Ramsden, G., & Botting, N. (2008). Emotional health in adolescents with and without a history of specific language impairment (SLI). The Journal of Child Psychology and Psychiatry, 49(5), 516–525. 10.1111/j.1469-7610.2007.01858.x 18221347

[bib33] Conti-Ramsden, G., Crutchley, A., & Botting, N. (1997). The extent to which psychometric tests differentiate subgroups of children with SLI. Journal of Speech, Language, and Hearing Research, 40(4), 765–777. 10.1044/jslhr.4004.765 9263942

[bib34] Courteau, É., Loignon, G., Steinhauer, K., & Royle, P. (2023). Identifying linguistic markers of French-speaking teenagers with developmental language disorder: Which tasks matter? Journal of Speech, Language, and Hearing Research, 66(1), 221–238. 10.1044/2022_JSLHR-21-00541 36599157

[bib35] Cuthbert, B. N. (2014). The RDoC framework: Facilitating transition from ICD/DSM to dimensional approaches that integrate neuroscience and psychopathology. World Psychiatry, 13(1), 28–35. 10.1002/wps.20087 24497240 PMC3918011

[bib36] Cuthbert, B. N. (2020). The role of RDoC in future classification of mental disorders. Dialogues in Clinical Neuroscience, 22(1), 81–85. 10.31887/DCNS.2020.22.1/bcuthbert 32699508 PMC7365298

[bib37] Deevy, P., Weil, L. W., Leonard, L. B., & Goffman, L. (2010). Extending use of the NRT to preschool-age children with and without specific language impairment. Language, Speech, and Hearing Services in Schools, 41(3), 277–288. 10.1044/0161-1461(2009/08-0096) 20421612 PMC2897931

[bib39] Dewey, D. (2018). What is comorbidity and why does it matter in neurodevelopmental disorders? Current Developmental Disorders Reports, 5(4), 235–242. 10.1007/s40474-018-0152-3

[bib38] De Wit, E., van Dijk, P., Hanekamp, S., Visser-Bochane, M. I., Steenbergen, B., van der Schans, C. P., & Luinge, M. R. (2018). Same or different: The overlap between children with auditory processing disorders and children with other developmental disorders: A systematic review. Ear and Hearing, 39(1), 1–19. 10.1097/AUD.0000000000000479 28863035 PMC7654752

[bib40] Dollaghan, C. A. (2004). Taxometric analyses of specific language impairment in 3- and 4-year-old children. Journal of Speech, Language, and Hearing Research, 47(2), 464–475. 10.1044/1092-4388(2004/037) 15157144

[bib41] Dollaghan, C. A. (2011). Taxometric analyses of specific language impairment in 6-year-old children. Journal of Speech, Language, and Hearing Research, 54(5), 1361–1371. 10.1044/1092-4388(2011/10-0187) 21646422

[bib42] Doucet, G. E., Kruse, J. A., Mertens, A., Goldsmith, C., Eden, N. M., Oleson, J., & McGregor, K. K. (2025). Subcortical brain iron and its link to verbal memory in children with developmental language disorder. Brain and Language, 261, Article 105531. 10.1016/j.bandl.2024.105531 PMC11769726 39756358

[bib43] Dubois, P., St-Pierre, M. C., Desmarais, C., & Guay, F. (2020). Young adults with developmental language disorder: A systematic review of education, employment, and independent living outcomes. Journal of Speech, Language, and Hearing Research, 63(11), 3786–3800. 10.1044/2020_JSLHR-20-00127 33022192

[bib44] Ebert, K. D., & Kohnert, K. (2011). Sustained attention in children with primary language impairment: A meta-analysis. Journal of Speech, Language, and Hearing Research, 54(5), 1372–1384. 10.1044/1092-4388(2011/10-0231) PMC4047633 21646419

[bib45] Eising, E., Mirza-Schreiber, N., De Zeeuw, E. L., Wang, C. A., Truong, D. T., Allegrini, A. G., Shapland, C. Y., Zhu, G., Wigg, K. G., Gerritse, M. L., Molz, B., Alagöz, G., Gialluisi, A., Abbondanza, F., Rimfeld, K., van Donkelaar, M., Liao, Z., Jansen, P. R., Andlauer, T. F. M., … Fisher, S. E. (2022). Genome-wide analyses of individual differences in quantitatively assessed reading- and language-related skills in up to 34,000 people. Proceedings of the National Academy of Sciences, 119(35), Article e2202764119. 10.1073/pnas.2202764119 PMC9436320 35998220

[bib46] Ellis Weismer, S., Tomblin, J. B., Durkin, M. S., Bolt, D., & Palta, M. (2021). A preliminary epidemiologic study of social (pragmatic) communication disorder in the context of developmental language disorder. International Journal of Language & Communication Disorders, 56(6), 1235–1248. 10.1111/1460-6984.12664 34383380 PMC8890438

[bib48] Evans, J. L., Saffran, J. R., & Robe-Torres, K. (2009). Statistical learning in children with specific language impairment. Journal of Speech, Language, and Hearing Research, 52(2), 321–335. 10.1044/1092-4388(2009/07-0189) PMC3864761 19339700

[bib49] Félix, J., Santos, M. E., & Benitez-Burraco, A. (2024). Specific language impairment, autism spectrum disorders and social (pragmatic) communication disorders: Is there overlap in language deficits? A review. Review Journal of Autism and Developmental Disorders, 11(1), 86–106. 10.1007/s40489-022-00327-5

[bib50] First, M. B. (2005). Mutually exclusive versus co-occurring diagnostic categories: The challenge of diagnostic comorbidity. Psychopathology, 38(4), 206–210. 10.1159/000086093 16145276

[bib51] Fiveash, A., Bedoin, N., Gordon, R. L., & Tillmann, B. (2021). Processing rhythm in speech and music: Shared mechanisms and implications for developmental speech and language disorders. Neuropsychology, 35(8), 771–791. 10.1037/neu0000766 34435803 PMC8595576

[bib52] Flapper, B. C., & Schoemaker, M. M. (2013). Developmental coordination disorder in children with specific language impairment: Co-morbidity and impact on quality of life. Research in Developmental Disabilities, 34(2), 756–763. 10.1016/j.ridd.2012.10.014 23220052

[bib53] Franck, J., Cronel-Ohayon, S., Chillier, L., Frauenfelder, U. H., Hamann, C., Rizzi, L., & Zesiger, P. (2004). Normal and pathological development of subject–verb agreement in speech production: A study on French children. Journal of Neurolinguistics, 17(2–3), 147–180. 10.1016/S0911-6044(03)00057-5

[bib54] Frazier, T. W., Youngstrom, E. A., Speer, L., Embacher, R., Law, P., Constantino, J., Findling, R. L., Hardan, A. Y., & Eng, C. (2012). Validation of proposed DSM-5 criteria for autism spectrum disorder. Journal of the American Academy of Child & Adolescent Psychiatry, 51(1), 28–40.e3. 10.1016/j.jaac.2011.09.021 22176937 PMC3244681

[bib55] Gallinat, E., & Spaulding, T. J. (2014). Differences in the performance of children with specific language impairment and their typically developing peers on nonverbal cognitive tests: A meta-analysis. Journal of Speech, Language, and Hearing Research, 57(4), 1363–1382. 10.1044/2014_JSLHR-L-12-0363 24686912

[bib56] Geng, L., & Sheng, L. (2025). Disfluency patterns and speech rate in Mandarin-speaking children with developmental language disorder: Evidence from narrative tasks [Paper presentation]. The 45th Annual Symposium on Research in Child Language Disorders, Madison, WI, United States.

[bib57] Gillberg, C. (2010). The ESSENCE in child psychiatry: Early Symptomatic Syndromes Eliciting Neurodevelopmental Clinical Examinations. Research in Developmental Disabilities, 31(6), 1543–1551. 10.1016/j.ridd.2010.06.002 20634041

[bib58] Goffman, L., Factor, L., Barna, M., Cai, F., & Feld, I. (2023). Phonological and articulatory deficits in the production of novel signs in children with developmental language disorder. Journal of Speech, Language, and Hearing Research, 66(3), 1051–1067. 10.1044/2022_JSLHR-22-00434 PMC10205102 36795546

[bib59] Goffman, L., & Gerken, L. (2023). A developmental account of the role of sequential dependencies in typical and atypical language learners. Cognitive Neuropsychology, 40(5–6), 243–264. 10.1080/02643294.2023.2275837 37963089 PMC10939949

[bib60] Graf, W. D. (2020). Beyond autism: Advocacy for neurodevelopmental differences. The American Journal of Bioethics, 20(4), 30–33. 10.1080/15265161.2020.1730503 32223626

[bib61] Graham, L. J. (2008). Drugs, labels and (p)ill-fitting boxes: ADHD and children who are hard to teach. Discourse: Studies in the Cultural Politics of Education, 29(1), 85–106. 10.1080/01596300701801377

[bib62] Graham, L. J., & Tancredi, H. (2019). In search of a middle ground: The dangers and affordances of diagnosis in relation to attention deficit hyperactivity disorder and developmental language disorder. Emotional and Behavioural Difficulties, 24(3), 287–300. 10.1080/13632752.2019.1609248

[bib63] Graham, S., Hebert, M., Fishman, E., Ray, A. B., & Rouse, A. G. (2020). Do children classified with specific language impairment have a learning disability in writing? A meta-analysis. Journal of Learning Disabilities, 53(4), 292–310. 10.1177/0022219420917338 32396037

[bib64] Grimm, A., & Schulz, P. (2014). Specific language impairment and early second language acquisition: The risk of over- and underdiagnosis. Child Indicators Research, 7(4), 821–841. 10.1007/s12187-013-9230-6

[bib65] Hall, J., McGregor, K. K., & Oleson, J. (2017). Weaknesses in lexical–semantic knowledge among college students with specific learning disabilities: Evidence from a semantic fluency task. Journal of Speech, Language, and Hearing Research, 60(3), 640–653. 10.1044/2016_JSLHR-L-15-0440 PMC5544191 28267833

[bib66] Hardman, G., Herman, R., Kyle, F. E., Ebbels, S., & Morgan, G. (2023). Identifying developmental language disorder in deaf children with cochlear implants: A case study of three children. Journal of Clinical Medicine, 12(17), Article 5755. 10.3390/jcm12175755 PMC10488728 37685824

[bib67] Hawa, V. V., & Spanoudis, G. (2014). Toddlers with delayed expressive language: An overview of the characteristics, risk factors and language outcomes. Research in Developmental Disabilities, 35(2), 400–407. 10.1016/j.ridd.2013.10.027 24334229

[bib68] Hawker, K., Ramirez-Inscoe, J., Bishop, D. V., Twomey, T., O'Donoghue, G. M., & Moore, D. R. (2008). Disproportionate language impairment in children using cochlear implants. Ear and Hearing, 29(3), 467–471. 10.1097/AUD.0b013e318167b857 18453886

[bib69] Hill, E. L. (2001). Non-specific nature of specific language impairment: A review of the literature with regard to concomitant motor impairments. International Journal of Language & Communication Disorders, 36(2), 149–171. 10.1080/13682820010019874 11344592

[bib70] Hobson, H. M., Toseeb, U., & Gibson, J. L. (2024). Developmental language disorder and neurodiversity: Surfacing contradictions, tensions and unanswered questions. International Journal of Language & Communication Disorders, 59(4), 1505–1516. 10.1111/1460-6984.13009 38275081

[bib71] Ibrahim, K., & Sukhodolsky, D. G. (2021). RDoC and autism. In F. R. Volkmar (Ed.), Encyclopedia of autism spectrum disorders (2nd ed., pp. 3840–3841). Springer Nature. 10.1044/2022_JSLHR-22-00221

[bib72] Individuals with Disabilities Education Improvement Act of 2004, Pub. L. No. 108–446,§118 Stat. 2647. (2004).

[bib73] Iverson, J. M. (2022). Developing language in a developing body, revisited: The cascading effects of motor development on the acquisition of language. Wiley Interdisciplinary Reviews: Cognitive Science, 13(6), Article e1626. 10.1002/wcs.1626 PMC12333486 36165333

[bib74] Jackson, E., Leitão, S., Claessen, M., & Boyes, M. (2020). Working, declarative, and procedural memory in children with developmental language disorder. Journal of Speech, Language, and Hearing Research, 63(12), 4162–4178. 10.1044/2020_JSLHR-20-00135 33237847

[bib75] Jackson, T., & Plante, E. (1996). Gyral morphology in the posterior Sylvian region in families affected by developmental language disorder. Neuropsychology Review, 6(2), 81–94. 10.1007/BF01875369 8976499

[bib76] Johnston, J. R., & Smith, L. B. (1989). Dimensional thinking in language-impaired children. Journal of Speech and Hearing Research, 32(1), 33–38. 10.1044/jshr.3201.33 2704199

[bib77] Kapa, L., Plante, E., & Doubleday, K. J. (2017). Applying an integrative framework of executive function to preschoolers with specific language impairment. Journal of Speech, Language, and Hearing Research, 60(8), 2170–2184. 10.1044/2017_JSLHR-L-16-0027 PMC5829800 28724132

[bib78] Karasik, L. B., Tamis-LeMonda, C. S., & Adolph, K. E. (2014). Crawling and walking infants elicit different verbal responses from mothers. Developmental Science, 17(3), 388–395. 10.1111/desc.12129 24314018 PMC3997624

[bib79] Karmiloff-Smith, A. (2006). The tortuous route from genes to behavior: A neuroconstructivist approach. Cognitive, Affective, & Behavioral Neuroscience, 6(1), 9–17. 10.3758/CABN.6.1.9 16869225

[bib80] Karmiloff-Smith, A. (2009). Nativism versus neuroconstructivism: Rethinking the study of developmental disorders. Developmental Psychology, 45(1), 56–63. 10.1037/a0014506 19209990

[bib81] Kim, Y. S., & State, M. W. (2014). Recent challenges to the psychiatric diagnostic nosology: A focus on the genetics and genomics of neurodevelopmental disorders. International Journal of Epidemiology, 43(2), 465–475. 10.1093/ije/dyu037 24618187 PMC4047292

[bib82] Kreidler, K., Vuolo, J., & Goffman, L. (2023). Children with developmental language disorder show deficits in the production of musical rhythmic groupings. Journal of Speech, Language, and Hearing Research, 66(11), 4481–4496. 10.1044/2023_JSLHR-23-00197 PMC10715845 37748025

[bib83] Krishnan, S., Cler, G. J., Smith, H. J., Willis, H. E., Asaridou, S. S., Healy, M. P., Papp, D., & Watkins, K. E. (2022). Quantitative MRI reveals differences in striatal myelin in children with DLD. eLife, 11, Article e74242. 10.7554/eLife.74242 PMC9514847 36164824

[bib84] Krishnan, S., Watkins, K. E., & Bishop, D. V. (2016). Neurobiological basis of language learning difficulties. Trends in Cognitive Sciences, 20(9), 701–714. 10.1016/j.tics.2016.06.012 27422443 PMC4993149

[bib85] Kuiack, A. K., & Archibald, L. M. (2024). Identifying and describing developmental language disorder in children. International Journal of Language & Communication Disorders, 59(3), 1180–1193. 10.1111/1460-6984.12984 38010314

[bib86] Kujala, T., & Leminen, M. (2017). Low-level neural auditory discrimination dysfunctions in specific language impairment—A review on mismatch negativity findings. Developmental Cognitive Neuroscience, 28, 65–75. 10.1016/j.dcn.2017.10.005 29182947 PMC6987907

[bib87] Kurth, F., Luders, E., Pigdon, L., Conti-Ramsden, G., Reilly, S., & Morgan, A. T. (2018). Altered gray matter volumes in language-associated regions in children with developmental language disorder and speech sound disorder. Developmental Psychobiology, 60(7), 814–824. 10.1002/dev.21762 30101474

[bib88] Lammertink, I., Boersma, P., Wijnen, F., & Rispens, J. (2017). Statistical learning in specific language impairment: A meta-analysis. Journal of Speech, Language, and Hearing Research, 60(12), 3474–3486. 10.1044/2017_JSLHR-L-16-0439 29149241

[bib89] Lancaster, H. S., & Camarata, S. (2019). Reconceptualizing developmental language disorder as a spectrum disorder: Issues and evidence. International Journal of Language & Communication Disorders, 54(1), 79–94. 10.1111/1460-6984.12433 30426606 PMC6684235

[bib90] Larson, C., & Ellis Weismer, S. (2022). Working memory performance in children with developmental language disorder: The role of domain. Journal of Speech, Language, and Hearing Research, 65(5), 1906–1920. 10.1044/2022_JSLHR-21-00420 PMC9559775 35394804

[bib91] Leonard, L. B. (1989). Language learnability and specific language impairment in children. Applied Psycholinguistics, 10(2), 179–202. 10.1017/S0142716400008511

[bib92] Leonard, L. B. (2000). Specific language impairment across languages. In D. V. M. Bishop & L. B. Leonard (Eds.), Speech and language impairments in children: Causes, characteristics, intervention and outcome (pp. 115–129). Psychology Press/Taylor & Francis.

[bib93] Leonard, L. B. (2017). Children with specific language impairment. MIT Press. 10.1093/acrefore/9780190236557.013.64

[bib94] Licari, M. K., Finlay-Jones, A., Reynolds, J. E., Alvares, G. A., Spittle, A. J., Downs, J., Whitehouse, A. J. O., Leonard, H., Evans, K. L., & Varcin, K. (2019). The brain basis of comorbidity in neurodevelopmental disorders. Current Developmental Disorders Reports, 6(1), 9–18. 10.1007/s40474-019-0156-7

[bib95] Marini, A., Piccolo, B., Taverna, L., Berginc, M., & Ozbič, M. (2020). The complex relation between executive functions and language in preschoolers with developmental language disorders. International Journal of Environmental Research and Public Health, 17(5), Article 1772. 10.3390/ijerph17051772 PMC7084239 32182903

[bib96] Marton, K., & Schwartz, R. G. (2003). Working memory capacity and language processes in children with specific language impairment. Journal of Speech, Language, and Hearing Research, 46(5), 1138–1153. 10.1044/1092-4388(2003/089) 14575348

[bib97] Mas, A., Lagadeuc, Y., & Vandenkoornhuyse, P. (2020). Reflections on the predictability of evolution: Toward a conceptual framework. iScience, 23(11), Article 101736. 10.1016/j.isci.2020.101736 PMC7666346 33225244

[bib98] Masten, A. S., & Cicchetti, D. (2010). Developmental cascades. Development and Psychopathology, 22(3), 491–495. 10.1017/S0954579410000222 20576173

[bib100] McGregor, K. K. (2020). How we fail children with developmental language disorder. Language, Speech, and Hearing Services in Schools, 51(4), 981–992. 10.1044/2020_LSHSS-20-00003 32755505 PMC7842848

[bib101] McGregor, K. K., Newman, R. M., Reilly, R. M., & Capone, N. C. (2002). Semantic representation and naming in children with specific language impairment. Journal of Speech, Language, and Hearing Research, 45(5), 998–1014. 10.1044/1092-4388(2002/081) 12381056

[bib102] McGregor, K. K., Pomper, R., Eden, N., Appenzeller, M., Arbisi-Kelm, T., Polese, E., & Reed, D. K. (2024). Inferring word class and meaning from spoken and written texts: A comparison of children with and without developmental language disorder. Journal of Speech, Language, and Hearing Research, 67(12), 4783–4798. 10.1044/2024_JSLHR-23-00743 PMC12379556 39589270

[bib103] McGregor, K. K., Sheng, L. I., & Smith, B. (2005). The precocious two-year-old: Status of the lexicon and links to the grammar. Journal of Child Language, 32(3), 563–585. 10.1017/S0305000905006926 16220635

[bib99] McKean, C., Wraith, D., Eadie, P., Cook, F., Mensah, F., & Reilly, S. (2017). Subgroups in language trajectories from 4 to 11 years: The nature and predictors of stable, improving and decreasing language trajectory groups. The Journal of Child Psychology and Psychiatry, 58(10), 1081–1091. 10.1111/jcpp.12790 28862345

[bib104] Miller, C. A., Kail, R., Leonard, L. B., & Tomblin, J. B. (2001). Speed of processing in children with specific language impairment. Journal of Speech, Language, and Hearing Research, 44(2), 416–433. 10.1044/1092-4388(2001/034) 11324662

[bib105] Mitchell, K. J. (2012). What is complex about complex disorders? Genome Biology, 13(1), Article 237. 10.1186/gb-2012-13-1-237 PMC3334577 22269335

[bib106] Montgomery, J. W. (2003). Working memory and comprehension in children with specific language impairment: What we know so far. Journal of Communication Disorders, 36(3), 221–231. 10.1016/s0021-9924(03)00021-2 12742669

[bib107] Mountford, H. S., Braden, R., Newbury, D. F., & Morgan, A. T. (2022). The genetic and molecular basis of developmental language disorder: A review. Children, 9(5), Article 586. 10.3390/children9050586 PMC9139417 35626763

[bib109] National Institute of Neurological Disorders and Stroke. (2024). Brain Basics: Genes and the brain. https://www.ninds.nih.gov/health-information/public-education/brain-basics/brain-basics-genes-and-brain

[bib108] Niemi, M. E., Martin, H. C., Rice, D. L., Gallone, G., Gordon, S., Kelemen, M., McAloney, K., McRae, J., Radford, E. J., Yu, S., Gecz, J., Martin, N. G., Wright, C. F., Fitzpatrick, D. R., Firth, H. V., Hurles, M. E., & Barrett, J. C. (2018). Common genetic variants contribute to risk of rare severe neurodevelopmental disorders. Nature, 562(7726), 268–271. 10.1038/s41586-018-0566-4 30258228 PMC6726472

[bib110] Nippold, M. A. (1985). Comprehension of figurative language in youth. Topics in Language Disorders, 5(3), 1–20. 10.1097/00011363-198506000-00004

[bib111] Nitin, R., Shaw, D. M., Rocha, D. B., Walters, C. E., Chabris, C. F., Camarata, S. M., Gordon, R. L., & Below, J. E. (2022). Association of developmental language disorder with comorbid developmental conditions using algorithmic phenotyping. JAMA Network Open, 5(12), Article e2248060. 10.1001/jamanetworkopen.2022.48060 PMC9857086 36580336

[bib112] Norbury, C. F. (2004). Factors supporting idiom comprehension in children with communication disorders. Journal of Speech, Language, and Hearing Research, 47(5), 1179–1193. 10.1044/1092-4388(2004/087) 15603470

[bib113] Norbury, C. F., Gooch, D., Wray, C., Baird, G., Charman, T., Simonoff, E., Vamvakas, G., & Pickles, A. (2016). The impact of nonverbal ability on prevalence and clinical presentation of language disorder: Evidence from a population study. The Journal of Child Psychology and Psychiatry, 57(11), 1247–1257. 10.1111/jcpp.12573 27184709 PMC5082564

[bib114] Norbury, C. F., Vamvakas, G., Gooch, D., Baird, G., Charman, T., Simonoff, E., & Pickles, A. (2017). Language growth in children with heterogeneous language disorders: A population study. The Journal of Child Psychology and Psychiatry, 58(10), 1092–1105. 10.1111/jcpp.12793 28921543 PMC5639364

[bib115] Obeid, R., Brooks, P. J., Powers, K. L., Gillespie-Lynch, K., & Lum, J. A. (2016). Statistical learning in specific language impairment and autism spectrum disorder: A meta-analysis. Frontiers in Psychology, 7, Article 1245. 10.3389/fpsyg.2016.01245 PMC4993848 27602006

[bib116] Parks, K. M., Hannah, K. E., Moreau, C. N., Brainin, L., & Joanisse, M. F. (2023). Language abilities in children and adolescents with DLD and ADHD: A scoping review. Journal of Communication Disorders, 106, Article 106381. 10.1016/j.jcomdis.2023.106381 37797400

[bib117] Pigdon, L., Willmott, C., Reilly, S., Conti-Ramsden, G., Gaser, C., Connelly, A., & Morgan, A. T. (2019). Grey matter volume in developmental speech and language disorder. Brain Structure and Function, 224(9), 3387–3398. 10.1007/s00429-019-01978-7 31732792

[bib119] Plante, E. (1991). MRI findings in the parents and siblings of specifically language-impaired boys. Brain and Language, 41(1), 67–80. 10.1016/0093-934X(91)90111-D 1884192

[bib118] Plante, E. (1998). Criteria for SLI: The Stark and Tallal legacy and beyond. Journal of Speech, Language, and Hearing Research, 41(4), 951–957. 10.1044/jslhr.4104.951 9712140

[bib120] Plante, E., Gomez, R., & Gerken, L. (2002). Sensitivity to word order cues by normal and language/learning disabled adults. Journal of Communication Disorders, 35(5), 453–462. 10.1016/S0021-9924(02)00094-1 12194564

[bib121] Plante, E., Patterson, D., Sandoval, M., Vance, C. J., & Asbjørnsen, A. E. (2017). An fMRI study of implicit language learning in developmental language impairment. NeuroImage: Clinical, 14, 277–285. 10.1016/j.nicl.2017.01.027 28203531 PMC5295640

[bib122] Plante, E., Ramage, A., & Maglöire, J. (2006). Processing narratives for verbatim and gist information by adults with language learning disabilities: A functional neuroimaging study. Learning Disabilities Research & Practice, 21(1), 61–76. 10.1111/j.1540-5826.2006.00207.x

[bib123] Plante, E., Swisher, L., Vance, R., & Rapcsak, S. (1991). MRI findings in boys with specific language impairment. Brain and Language, 41(1), 52–66. 10.1016/0093-934X(91)90110-M 1884191

[bib124] Plante, E., Vance, R., Moody, A., & Gerken, L. A. (2013). What influences children's conceptualizations of language input? Journal of Speech, Language, and Hearing Research, 56(5), 1613–1624. 10.1044/1092-4388(2013/12-0129) 23785193

[bib125] Preza, T., Liu, D., Miller, C., Alabdallah, A., Xiong, J., Redmond, S., & Hadley, P. (2025). Characterizing school-age children's effort and automaticity in a sentence recall task [Poster presentation]. Symposium on Research in Child Language Disorders, Madison, WI, United States.

[bib126] Ramirez-Inscoe, J., & Moore, D. R.(2011). Processes that influence communicative impairments in deaf children using cochlear implants. Ear and Hearing, 32(6), 690–698. 10.1097/AUD.0b013e31821f0538 21637101

[bib127] Redmond, S. M., Thompson, H. L., & Goldstein, S. (2011). Psycholinguistic profiling differentiates specific language impairment from typical development and from attention-deficit/hyperactivity disorder. Journal of Speech, Language, and Hearing Research, 54(1), 99–117. 10.1044/1092-4388(2010/10-0010) PMC4493886 20719871

[bib128] Reilly, S., Tomblin, B., Law, J., McKean, C., Mensah, F. K., Morgan, A., Goldfeld, S., Nicholson, J. M., & Wake, M. (2014). Specific language impairment: A convenient label for whom? International Journal of Language & Communication Disorders, 49(4), 416–451. 10.1111/1460-6984.12102 25142091 PMC4303922

[bib129] Rice, M. L. (2013). Language growth and genetics of specific language impairment. International Journal of Speech-Language Pathology, 15(3), 223–233. 10.3109/17549507.2013.783113 23614332 PMC3684183

[bib171] Rice, M. L., & Hoffman, L. (2015). Predicting vocabulary growth in children with and without specific language impairment: A longitudinal study from 2; 6 to 21 years of age. Journal of Speech, Language, and Hearing Research, 58(2), 345–359. 10.1044/2015_JSLHR-L-14-0150 PMC4398600 25611623

[bib130] Rice, M. L., Hoffman, L., & Wexler, K. (2009). Judgments of omitted BE and DO in questions as extended finiteness clinical markers of specific language impairment (SLI) to 15 years: A study of growth and asymptote. Journal of Speech, Language, and Hearing Research, 52(6), 1417–1433. 10.1044/1092-4388(2009/08-0171) PMC2787761 19786705

[bib131] Rice, M. L., Warren, S. F., & Betz, S. K. (2005). Language symptoms of developmental language disorders: An overview of autism, down syndrome, fragile X, specific language impairment, and Williams syndrome. Applied Psycholinguistics, 26(1), 7–27. 10.1017/S0142716405050034

[bib132] Rice, M. L., & Wexler, K. (1996). Toward tense as a clinical marker of specific language impairment in English-speaking children. Journal of Speech and Hearing Research, 39(6), 1239–1257. 10.1044/jshr.3906.1239 8959609

[bib133] Rujas, I., Mariscal, S., Murillo, E., & Lázaro, M. (2021). Sentence repetition tasks to detect and prevent language difficulties: A scoping review. Children, 8(7), Article 578. 10.3390/children8070578 PMC8305617 34356557

[bib134] Sack, L., Dollaghan, C., & Goffman, L. (2022). Contributions of early motor deficits in predicting language outcomes among preschoolers with developmental language disorder. International Journal of Speech-Language Pathology, 24(4), 362–374. 10.1080/17549507.2021.1998629 34793281 PMC9881565

[bib47] Sanchez, A., Long, B., Kraus, A. M., & Frank, M. C. (2018). Postural developments modulate children's visual access to social information. In Proceedings of the 40th Annual Conference of the Cognitive Science Society (pp. 2412–2417). Cognitive Science Society. 10.31234/osf.io/th92b

[bib135] Sapiets, S. J. (2021). Embracing complexity in research on neurodevelopmental conditions and mental health. Embracing Complexity. https://kar.kent.ac.uk/93151/1/Embracing-Complexity-in-Research-on-Neurodevelopmental-Conditions.pdf [PDF]

[bib136] Scott, C. M., & Windsor, J. (2000). General language performance measures in spoken and written narrative and expository discourse of school-age children with language learning disabilities. Journal of Speech, Language, and Hearing Research, 43(2), 324–339. 10.1044/jslhr.4302.324 10757687

[bib137] Shriberg, L. D., Tomblin, J. B., & McSweeny, J. L. (1999). Prevalence of speech delay in 6-year-old children and comorbidity with language impairment. Journal of Speech, Language, and Hearing Research, 42(6), 1461–1481. 10.1044/jslhr.4206.1461 10599627

[bib138] Simpson, K., Paynter, J., Ziegenfusz, S., & Westerveld, M. (2022). Sensory profiles in school-age children with developmental language disorder. International Journal of Disability, Development and Education, 69(3), 781–790. 10.1080/1034912X.2020.1740186

[bib139] Sirois, S., Spratling, M., Thomas, M. S. C., Westermann, G., Mareschal, D., & Johnson, M. H. (2008). Précis of neuroconstructivism: How the brain constructs cognition. Behavioral and Brain Sciences, 31(3), 321–331. 10.1017/S0140525X0800407X 18578929

[bib140] Smolak, E., McGregor, K. K., Arbisi-Kelm, T., & Eden, N. (2020). Sustained attention in developmental language disorder and its relation to working memory and language. Journal of Speech, Language, and Hearing Research, 63(12), 4096–4108. 10.1044/2020_JSLHR-20-00265 PMC8608174 33166200

[bib142] Sonuga-Barke, E. J. (1998). Categorical models of childhood disorder: A conceptual and empirical analysis. The Journal of Child Psychology and Psychiatry, 39(1), 115–133. 10.1111/1469-7610.00306 9534089

[bib143] Spaulding, T. J., Plante, E., & Vance, R. B. (2008). Sustained selective attention skills of preschool children with specific language impairment: Evidence for separate attentional capacities. Journal of Speech, Language, and Hearing Research, 51(1), 16–34. 10.1044/1092-4388(2008/002) 18230853

[bib144] Stark, R. E., & Tallal, P. (1981). Selection of children with specific language deficits. Journal of Speech and Hearing Disorders, 46(2), 114–122. 10.1044/jshd.4602.114 7253588

[bib146] Thordardottir, E. T., & Namazi, M. (2007). Specific language impairment in French-speaking children: Beyond grammatical morphology. Journal of Speech, Language, and Hearing Research, 50(3), 698–715. 10.1044/1092-4388(2007/049) 17538110

[bib147] Tighe, J. M., & Namazi, M. (2022). SPICES: Disclosure practices to help caregivers digest a diagnosis of developmental language disorder. American Journal of Speech-Language Pathology, 31(5), 1919–1932. 10.1044/2022_AJSLP-21-00295 36007195

[bib148] Tomblin, J. B., Records, N. L., Buckwalter, P., Zhang, X., Smith, E., & O'Brien, M. (1997). Prevalence of specific language impairment in kindergarten children. Journal of Speech, Language, and Hearing Research, 40(6), 1245–1260. 10.1044/jslhr.4006.1245 PMC5075245 9430746

[bib149] Tomblin, J. B., & Zhang, X. (2006). The dimensionality of language ability in school-age children. Journal of Speech, Language, and Hearing Research, 49(6), 1193–1208. 10.1044/1092-4388(2006/086) 17197490

[bib150] Tomblin, J. B., Zhang, X., Buckwalter, P., & Catts, H. (2000). The association of reading disability, behavioral disorders, and language impairment among second-grade children. The Journal of Child Psychology and Psychiatry, 41(4), 473–482. 10.1111/1469-7610.00632 10836677

[bib151] Tomblin, J. B., Zhang, X., Buckwalter, P., & O'Brien, M. (2003). The stability of primary language disorder: Four years after kindergarten diagnosis. Journal of Speech, Language, and Hearing Research, 46(6), 1283–1296. 10.1044/1092-4388(2003/100) 14700355

[bib152] Ullman, M. T., Clark, G. M., Pullman, M. Y., Lovelett, J. T., Pierpont, E. I., Jiang, X., & Turkeltaub, P. E. (2024). The neuroanatomy of developmental language disorder: A systematic review and meta-analysis. Nature Human Behavior, 8(5), 962–975. 10.1038/s41562-024-01843-6 38491094

[bib153] Ullman, M. T., Earle, F. S., Walenski, M., & Janacsek, K. (2020). The neurocognition of developmental disorders of language. Annual Review of Psychology, 71(1), 389–417. 10.1146/annurev-psych-122216-011555 31337273

[bib154] Ullman, M. T., & Pierpont, E. I. (2005). Specific language impairment is not specific to language: The procedural deficit hypothesis. Cortex, 41(3), 399–433. 10.1016/S0010-9452(08)70276-4 15871604

[bib155] van Daal, J., Verhoeven, L., & van Balkom, H. (2004). Subtypes of severe speech and language impairments: Psychometric evidence from 4-year-old children in the Netherlands. Journal of Speech, Language, and Hearing Research, 47(6), 1411–1423. 10.1044/1092-4388(2004/105) 15842019

[bib156] van der Lely, H. K., & Pinker, S. (2014). The biological basis of language: Insight from developmental grammatical impairments. Trends in Cognitive Sciences, 18(11), 586–595. 10.1016/j.tics.2014.07.001 25172525

[bib157] van Dijk, M., van Geert, P., Korecky-Kröll, K., Maillochon, I., Laaha, S., Dressler, W. U., & Bassano, D. (2013). Dynamic adaptation in child–adult language interaction. Language Learning, 63(2), 243–270. 10.1111/lang.12002

[bib158] Van Weerdenburg, M., Verhoeven, L., & van Balkom, H. (2006). Towards a typology of specific language impairment. The Journal of Child Psychology and Psychiatry, 47(2), 176–189. 10.1111/j.1469-7610.2005.01454.x 16423149

[bib159] van Wijngaarden, V., de Wilde, H., Mink van der Molen, D., Petter, J., Stegeman, I., Gerrits, E., Smit, A. L., & van den Boogaard, M. J. (2024). Genetic outcomes in children with developmental language disorder: A systematic review. Frontiers in Pediatrics, 12, Article 1315229. 10.3389/fped.2024.1315229 PMC10828955 38298611

[bib160] Walle, E. A., & Campos, J. J. (2014). Infant language development is related to the acquisition of walking. Developmental Psychology, 50(2), 336–348. 10.1037/a0033238 23750505

[bib161] Weckerly, J., Wulfeck, B., & Reilly, J. (2001). Verbal fluency deficits in children with specific language impairment: Slow rapid naming or slow to name? Child Neuropsychology, 7(3), 142–152. 10.1076/chin.7.3.142.8741 12187471

[bib162] Werfel, K. L., Hendricks, A. E., & Schuele, C. M. (2017). The potential of past tense marking in oral reading as a clinical marker of specific language impairment in school-age children. Journal of Speech, Language, and Hearing Research, 60(12), 3561–3572. 10.1044/2017_JSLHR-L-17-0115 PMC6111522 29222571

[bib163] West, G., Vadillo, M. A., Shanks, D. R., & Hulme, C. (2018). The procedural learning deficit hypothesis of language learning disorders: We see some problems. Developmental Science, 21(2), Article e12552. 10.1111/desc.12552 PMC5888158 28256101

[bib164] Westerlund, M., Bergkvist, L., Lagerberg, D., & Sundelin, C. (2002). Comorbidity in children with severe developmental language disability. Acta Paediatrica, 91(5), 529–534. 10.1111/j.1651-2227.2002.tb03272.x 12113321

[bib165] Williams, D., Botting, N., & Boucher, J. (2008). Language in autism and specific language impairment: Where are the links? Psychological Bulletin, 134(6), 944–963. 10.1037/a0013743 18954162

[bib166] Windsor, J., & Hwang, M. (1999). Testing the generalized slowing hypothesis in specific language impairment. Journal of Speech, Language, and Hearing Research, 42(5), 1205–1218. 10.1044/jslhr.4205.1205 10515516

[bib167] Wolfus, B., Moscovitch, M., & Kinsbourne, M. (1980). Subgroups of developmental language impairment. Brain and Language, 10(1), 152–171. 10.1016/0093-934X(80)90046-2 7378721

[bib168] World Health Organization. (2022). International Classification of Diseases–Eleventh Revision (ICD-11).

[bib169] Young, A. R., Beitchman, J. H., Johnson, C., Douglas, L., Atkinson, L., Escobar, M., & Wilson, B. (2002). Young adult academic outcomes in a longitudinal sample of early identified language impaired and control children. The Journal of Child Psychology and Psychiatry, 43(5), 635–645. 10.1111/1469-7610.00052 12120859

[bib170] Zapparrata, N. M., Brooks, P. J., & Ober, T. (2023). Developmental language disorder is associated with slower processing across domains: A meta-analysis of time-based tasks. Journal of Speech, Language, and Hearing Research, 66(1), 325–346. 10.1044/2022_JSLHR-22-00221 36603228

